# Supplementary material for: A comparative study of postnatal anthropometric growth in very preterm infants and intrauterine growth
Source: Nat Commun. 2023 Sep 19;14:5626. doi: 10.1038/s41467-023-41069-0 (PMC10509139; doi:10.1038/s41467-023-41069-0)

Supplemental Data

Supplemental data for the manuscript entitled "A Comparative Study of Postnatal Anthropometric Growth in Very Preterm Infants and Intrauterine Growth".

Table of Content:

Table S1. Demographic characteristics of infants by gestational age group and sex................. 1
Table S2. Infant mortality and morbidities by gestational age group and sex 2
Table S3. The percentage of measurement values above or below the indicated percentile lines of the 2013 Fenton growth charts... 4
Table S4. The percentage of outliers for each nearest gestational age group 5
Table S5. The percentage of measurement values above or below the indicated percentile lines of the postnatal growth charts 6
Figures S1-S8. Comparing postnatal weight, length, and head circumference models for each of the gestational age groups to the 2013 Fenton growth charts. 7-14
Figure S9. Marginal mean absolute error (MAE) and root-mean-square error (RMSE) comparison between the training and the validation datasets..15
Figure S10. R-squared comparison between the training and the validation datasets. 16
Figures S11-S18. Comparing postnatal weight, length, and head circumference growth rates of each of the gestational age group to the intrauterine growth rates 17-24
Figures S19-S25. Percentile-specific growth rates during the weight acceleration and the stable weight gain phases. 25-31
Supplemental Figure S26. Sex differences in growth trajectory estimates................................32

Table S1. Demographic characteristics of infants by gestational age group and sex

Gestational Age Group	23	24	25	26	27	28	29	30
**Birth Gestational Age Range**	**22w4d-23w3d**	**23w4d-24w3d**	**24w4d-25w3d**	**25w4d-26w3d**	**26w4d-27w3d**	**27w4d-28w3d**	**28w4d-29w3d**	**29w4d-30w3d**
**Female Infants**								
**Number of Infants**	1505	3214	4008	4646	5412	6568	7432	9013
**Number of Data points**								
Weight	84095	223223	302588	350544	384065	414624	415382	427487
Length	11601	30198	40378	46869	52763	56461	58333	62365
Head Circumference	22312	57025	73519	83690	90108	95593	98289	99543
**Race/Ethnicity**								
White	491 (33%)	1022 (32%)	1475 (37%)	1732 (37%)	2224 (41%)	2715 (41%)	3208 (43%)	4063 (45%)
Black	550 (37%)	1175 (37%)	1318 (33%)	1573 (34%)	1633 (30%)	1915 (29%)	2110 (28%)	2263 (25%)
Hispanic	320 (21%)	704 (22%)	782 (20%)	881 (19%)	981 (18%)	1170 (18%)	1281 (17%)	1653 (18%)
Asian	32 (2%)	76 (2%)	131 (3%)	130 (3%)	146 (3%)	194 (3%)	207 (3%)	263 (3%)
Other	112 (7%)	237 (7%)	302 (8%)	330 (7%)	428 (8%)	574 (9%)	626 (8%)	771 (9%)
**Multiple Gestation**	335 (22%)	635 (20%)	878 (22%)	1039 (22%)	1246 (23%)	1610 (25%)	1903 (26%)	2479 (28%)
**Birth Weight (gram)**	544±76	607±98	690±127	788±151	896±184	1021±203	1159±226	1314±245
**Cesarean Delivery**	720 (48%)	2085 (65%)	2858 (71%)	3315 (71%)	4043 (75%)	4890 (74%)	5460 (73%)	6671 (74%)
**Male Infants**								
**Number of Infants**	1677	3752	4692	5175	6218	7278	8291	10337
**Number of Data points**								
Weight	76871	235496	335722	376655	427707	463333	468571	491692
Length	10756	31408	45020	50776	57885	63674	66570	70630
Head Circumference	20951	59756	83665	89993	103469	108434	111839	117901
**Race/Ethnicity**								
White	515 (31%)	1339 (36%)	1752 (37%)	1972 (38%)	2450 (39%)	3164 (43%)	3734 (45%)	4772 (46%)
Black	612 (36%)	1220 (33%)	1472 (31%)	1557 (30%)	1814 (29%)	1906 (26%)	2054 (25%)	2405 (23%)
Hispanic	387 (23%)	796 (21%)	962 (21%)	1030 (20%)	1256 (20%)	1353 (19%)	1521 (18%)	1931 (19%)
Asian	33 (2%)	107 (3%)	135 (3%)	159 (3%)	203 (3%)	239 (3%)	273 (3%)	315 (3%)
Other	130 (8%)	290 (8%)	371 (8%)	457 (9%)	495 (8%)	616 (8%)	709 (9%)	914 (9%)
**Multiple Gestation**	378 (23%)	798 (21%)	891 (19%)	1047 (20%)	1412 (23%)	1715 (24%)	2013 (24%)	2606 (25%)
**Birth Weight (gram)**	577±83	652±101	738±127	841±156	963±186	1087±210	1234±231	1383±259
**Cesarean Delivery**	813 (49%)	2416 (64%)	3256 (69%)	3696 (71%)	4427 (71%)	5200 (71%)	5939 (72%)	7372 (71%)

DOL: day of life
Count data are presented as number (percentage). Measurement values are presented as mean±sd.

Table S2. Infant mortality and morbidities by gestational age group and sex

Gestational age group	23	24	25	26	27	28	29	30
Birth gestational age range	22w4d-23w3d	23w4d-24w3d	24w4d-25w3d	25w4d-26w3d	26w4d-27w3d	27w4d-28w3d	28w4d-29w3d	29w4d-30w3d
FEMALE INFANTS								
**Discharge Status**								
Home	525 (35%)	1616 (50%)	2479 (62%)	3380 (73%)	4302 (79%)	5534 (84%)	6476 (87%)	8079 (90%)
Transfer to another facility	302 (20%)	616 (19%)	790 (20%)	755 (16%)	733 (14%)	762 (12%)	742 (10%)	744 (8%)
Deceased	678 (45%)	982 (31%)	739 (18%)	511 (11%)	377 (7%)	272 (4%)	214 (3%)	190 (2%)
**Major anomalies**	215 (14%)	521 (16%)	600 (15%)	638 (14%)	715 (13%)	759 (12%)	789 (11%)	937 (10%)
**IVH**								
Any	777 (52%)	1592 (50%)	1669 (42%)	1556 (33%)	1421 (26%)	1436 (22%)	1482 (20%)	1641 (18%)
Grade 3/4	364 (24%)	713 (22%)	605 (15%)	453 (10%)	325 (6%)	258 (4%)	213 (3%)	139 (2%)
**NEC**								
Medical	63 (4.19%)	130 (4.04%)	186 (4.64%)	197 (4.24%)	180 (3.33%)	212 (3.23%)	193 (2.60%)	208 (2.31%)
Surgical	60 (3.99%)	116 (3.61%)	122 (3.04%)	137 (2.95%)	82 (1.52%)	71 (1.08%)	44 (0.59%)	45 (0.50%)
**Late-Onset Sepsis**								
More than 3 days of Antibiotics	896 (60%)	2184 (68%)	2755 (69%)	2771 (60%)	2593 (48%)	2456 (37%)	2078 (28%)	1868 (21%)
Positive Blood Culture	407 (27%)	814 (25%)	918 (23%)	812 (17%)	667 (12%)	599 (9%)	472 (6%)	342 (4%)
**BPD***								
Any Grade	520 (35%)	1452 (45%)	1907 (48%)	2051 (44%)	2005 (37%)	1697 (26%)	1335 (18%)	1051 (12%)
Grade 2 or 3	348 (23%)	855 (27%)	1034 (26%)	1046 (23%)	895 (17%)	613 (9%)	501 (7%)	303 (3%)
**ROP**								
Any Stage	563 (37%)	1494 (46%)	2038 (51%)	2118 (46%)	1980 (37%)	1687 (26%)	1402 (19%)	929 (10%)
Higher than Stage 1	434 (29%)	1097 (34%)	1319 (33%)	1147 (25%)	855 (16%)	549 (8%)	378 (5%)	204 (2%)
Treated	130 (8.64%)	298 (9.27%)	316 (7.88%)	199 (4.28%)	84 (1.55%)	50 (0.76%)	29 (0.39%)	14 (0.16%)
**PVL**								
Any Reported	82 (5.45%)	180 (5.60%)	197 (4.92%)	200 (4.30%)	183 (3.38%)	179 (2.73%)	147 (1.98%)	122 (1.35%)
**Steroid Exposure**								
Antenatal	961 (64%)	2466 (77%)	3219 (80%)	3846 (83%)	4506 (83%)	5522 (84%)	6270 (84%)	7585 (84%)
Postnatal	355 (24%)	932 (29%)	995 (25%)	813 (17%)	487 (9%)	309 (5%)	201 (3%)	100 (1%)
MALE INFANTS								
**Discharge Status**								
Home	443 (26%)	1625 (43%)	2708 (58%)	3499 (68%)	4712 (76%)	5947 (82%)	7194 (87%)	9175 (89%)
Transfer to another facility	336 (20%)	813 (22%)	973 (21%)	933 (18%)	996 (16%)	938 (13%)	810 (10%)	922 (9%)
Deceased	898 (54%)	1314 (35%)	1011 (21%)	743 (14%)	510 (8%)	393 (5%)	287 (3%)	240 (2%)
**Major anomalies**	235 (14%)	581 (15%)	800 (17%)	818 (16%)	917 (15%)	991 (14%)	1030 (12%)	1167 (11%)

**IVH**								
Any	921 (55%)	1939 (52%)	2091 (45%)	2001 (39%)	1966 (32%)	1871 (26%)	1810 (22%)	1982 (19%)
Grade 3/4	507 (30%)	979 (26%)	891 (19%)	698 (13%)	570 (9%)	403 (6%)	282 (3%)	231 (2%)
**NEC**								
Medical	49 (2.92%)	175 (4.66%)	227 (4.84%)	237 (4.58%)	230 (3.70%)	265 (3.64%)	259 (3.12%)	211 (2.04%)
Surgical	65 (3.88%)	163 (4.34%)	152 (3.24%)	154 (2.98%)	112 (1.80%)	92 (1.26%)	83 (1.00%)	60 (0.58%)
Late-Onset Sepsis								
More than 3 days of Antibiotics	928 (55%)	2494 (66%)	3228 (69%)	3355 (65%)	3356 (54%)	3113 (43%)	2764 (33%)	2528 (24%)
Positive Blood Culture	411 (25%)	995 (27%)	1079 (23%)	1001 (19%)	889 (14%)	745 (10%)	562 (7%)	478 (5%)
**BPD***								
Any Grade	480 (29%)	1605 (43%)	2301 (49%)	2406 (46%)	2548 (41%)	2322 (32%)	1960 (24%)	1656 (16%)
Grade 2 or 3	312 (19%)	1030 (27%)	1328 (28%)	1312 (25%)	1231 (20%)	999 (14%)	730 (9%)	552 (5%)
**ROP**								
Any Stage	481 (29%)	1599 (43%)	2240 (48%)	2234 (43%)	2104 (34%)	1739 (24%)	1329 (16%)	931 (9%)
Higher than Stage 1	395 (24%)	1199 (32%)	1529 (33%)	1295 (25%)	964 (16%)	588 (8%)	413 (5%)	204 (2%)
Treated	150 (9%)	417 (11%)	388 (8%)	194 (4%)	139 (2%)	46 (0.64%)	30 (0.36%)	17 (0.16%)
**PVL**								
Any Reported	91 (5%)	219 (6%)	282 (6%)	244 (5%)	223 (4%)	209 (3%)	195 (2%)	178 (2%)
**Steroid Exposure**								
Antenatal	1080 (64%)	2919 (78%)	3732 (80%)	4197 (81%)	5126 (82%)	6008 (83%)	6994 (84%)	8574 (83%)
Postnatal	401 (24%)	1116 (30%)	1316 (28%)	1035 (20%)	756 (12%)	551 (8%)	327 (4%)	192 (2%)

IVH: intraventricular hemorrhage. NEC: necrotizing enterocolitis. BPD: bronchopulmonary dysplasia. ROP: retinopathy of prematurity. PVL: periventricular leukomalacia.
Data are presented as number (percentage).
*Based on the NICHD Neonatal Research Network grading system (Ref 10).

Table S3. The percentage of measurement values above or below the indicated percentile lines of the 2013 Fenton growth charts

Gestational age group	23	24	25	26	27	28	29	30
**Gestational age of measurement data**	**23w0d**	**24w0d**	**25w0d**	**26w0d**	**27w0d**	**28w0d**	**29w0d**	**30w0d**
**Female**								
**Weight**								
> 90th percentile line (%)	1.0	0.6	0.5	0.3	0.4	0.7	0.5	0.4
< 50th percentile line (%)	88.1	89.0	89.6	87.6	88.4	87.9	87.2	86.5
< 10th percentile line (%)	34.3	30.9	30.4	27.4	30.2	27.9	24.6	27.2
Between 10th and 90th percentile line (%)	64.7	68.5	69.1	72.3	69.4	71.4	74.9	72.4
**Length**								
> 90th percentile line (%)		1.3	1.5	1.4	1.8	2.5	2.9	3.3
< 50th percentile line (%)		87.8	87.2	84.2	82.9	78.1	73.9	70.6
< 10th percentile line (%)		50.2	49.8	43.1	39.7	32.7	27.5	25.6
Between 10th and 90th percentile line (%)		48.5	48.7	55.5	58.5	64.8	69.6	71.1
**Head Circumference**								
> 90th percentile line (%)		1.9	1.5	1.9	2.0	3.3	2.8	2.4
< 50th percentile line (%)		89.1	89.1	87.6	87.0	82.1	80.0	76.2
< 10th percentile line (%)		52.1	51.8	45.1	43.6	35.8	28.0	25.4
Between 10th and 90th percentile line (%)		46.0	46.7	53.0	54.4	60.9	69.2	72.2
**Male**								
**Weight**								
> 90th percentile line (%)	1.3	1.5	1.4	1.8	2.5	2.9	3.3	1.3
< 50th percentile line (%)	87.8	87.2	84.2	82.9	78.1	73.9	70.6	87.8
< 10th percentile line (%)	50.2	49.8	43.1	39.7	32.7	27.5	25.6	50.2
Between 10th and 90th percentile line (%)	48.5	48.7	55.5	58.5	64.8	69.6	71.1	48.5
**Length**								
> 90th percentile line (%)		1.1	0.9	1.5	1.4	1.8	2.8	3.5
< 50th percentile line (%)		89.1	88.4	86.0	81.5	81.2	74.5	72.4
< 10th percentile line (%)		54.3	50.3	46.8	39.9	37.1	28.7	29.0
Between 10th and 90th percentile line (%)		44.6	48.8	51.7	58.7	61.1	68.5	67.5
**Head Circumference**								
> 90th percentile line (%)		1.6	1.1	2.0	2.1	2.3	2.8	2.6
< 50th percentile line (%)		88.4	88.8	85.8	83.9	80.7	75.8	76.7
< 10th percentile line (%)		56.7	51.9	44.3	40.6	33	24.4	23.2
Between 10th and 90th percentile line (%)		41.7	47.0	53.7	57.3	64.7	72.8	74.2

Table S4. The percentage of outliers for each nearest gestational age group

Gestational age group	23	24	25	26	27	28	29	30
**Birth gestational age range**	**22w4d-23w3d**	**23w4d-24w3d**	**24w4d-25w3d**	**25w4d-26w3d**	**26w4d-27w3d**	**27w4d-28w3d**	**28w4d-29w3d**	**29w4d-30w3d**
**Female**								
**Weight**	0.03%	0.04%	0.03%	0.03%	0.03%	0.03%	0.04%	0.04%
**Length**	0.05%	0.08%	0.06%	0.03%	0.04%	0.04%	0.02%	0.01%
**Head Circumference**	0.13%	0.07%	0.06%	0.08%	0.07%	0.05%	0.05%	0.03%
**Male**								
**Weight**	0.03%	0.04%	0.03%	0.04%	0.03%	0.04%	0.03%	0.02%
**Length**	0.09%	0.08%	0.03%	0.04%	0.05%	0.04%	0.03%	0.02%
**Head Circumference**	0.11%	0.07%	0.10%	0.08%	0.08%	0.06%	0.04%	0.05%
Outliers are defined as leverage > 3 times mean leverage AND absolute standardized residual > 2.								

Table S5. The percentage of measurement values above or below the indicated percentile lines of the postnatal growth charts

Gestational age group	23	24	25	26	27	28	29	30
**Gestational age of measurement data**	**23w0d**	**24w0d**	**25w0d**	**26w0d**	**27w0d**	**28w0d**	**29w0d**	**30w0d**
**Female**								
**Weight**								
> 90th percentile line (%)	5.6	6.0	7.5	7.4	7.0	7.5	7.2	7.3
< 50th percentile line (%)	54.2	53.4	52.3	51.8	51.4	52.2	52.0	52.2
< 10th percentile line (%)	6.2	7.3	8.7	10.4	10.8	11.1	11.3	11.5
Between 10th and 90th percentile line (%)	88.2	86.7	83.8	82.2	82.2	81.4	81.5	81.2
**Length**								
> 90th percentile line (%)	5.7	5.7	5.0	4.6	4.1	4.5	4.5	4.7
< 50th percentile line (%)	54.8	56.5	55.7	54.8	55.5	55.1	54.7	55.3
< 10th percentile line (%)	9.6	11.6	11.4	12.4	12.5	12.9	12.6	13.1
Between 10th and 90th percentile line (%)	84.7	82.7	83.6	83.0	83.4	82.6	82.9	82.2
**Head Circumference**								
> 90th percentile line (%)	5.0	4.7	4.3	3.9	3.7	4.4	4.2	4.0
< 50th percentile line (%)	63.1	61.5	63.2	62.4	61.7	61.6	60.9	62.5
< 10th percentile line (%)	12.0	12.7	13.7	13.4	13.7	15.1	14.7	14.9
Between 10th and 90th percentile line (%)	83.0	82.6	82.0	82.7	82.6	80.5	81.1	81.1
**Male**								
**Weight**								
> 90th percentile line (%)	5.5	6.3	7.5	7.5	7.7	7.2	7.1	7.1
< 50th percentile line (%)	53.6	55.0	51.8	51.5	51.2	51.1	51.3	51.9
< 10th percentile line (%)	6.7	7.3	8.9	9.7	10.7	11.1	11.1	11.6
Between 10th and 90th percentile line (%)	87.8	86.4	83.6	82.8	81.6	81.7	81.8	81.3
**Length**								
> 90th percentile line (%)	5.5	5.4	5.4	5.0	4.3	4.5	5.0	5.0
< 50th percentile line (%)	54.2	54.9	53.5	54.2	52.7	54.4	54.1	54.8
< 10th percentile line (%)	10.8	10.6	11.4	11.5	11.8	12.5	13.1	12.5
Between 10th and 90th percentile line (%)	83.7	84.0	83.2	83.5	83.9	83.0	81.9	82.5
**Head Circumference**								
> 90th percentile line (%)	4.5	4.8	5.0	4.7	4.2	4.3	4.0	4.1
< 50th percentile line (%)	61.5	61.9	61.3	62.9	61.2	60.5	64.1	63.4
< 10th percentile line (%)	12.4	13.3	13.1	13.1	14.0	15.0	14.6	14.5
Between 10th and 90th percentile line (%)	83.1	81.9	81.9	82.2	81.8	80.7	81.4	81.4

Figure S1. Postnatal and intrauterine growth comparison for the 23-week gestational age group. Comparing postnatal weight (a-c,j-l), length (d-f,m-o), and head circumference (g-i,p-r) models for male (a-i) and female (j-r) infants to the 2013 Fenton growth charts.

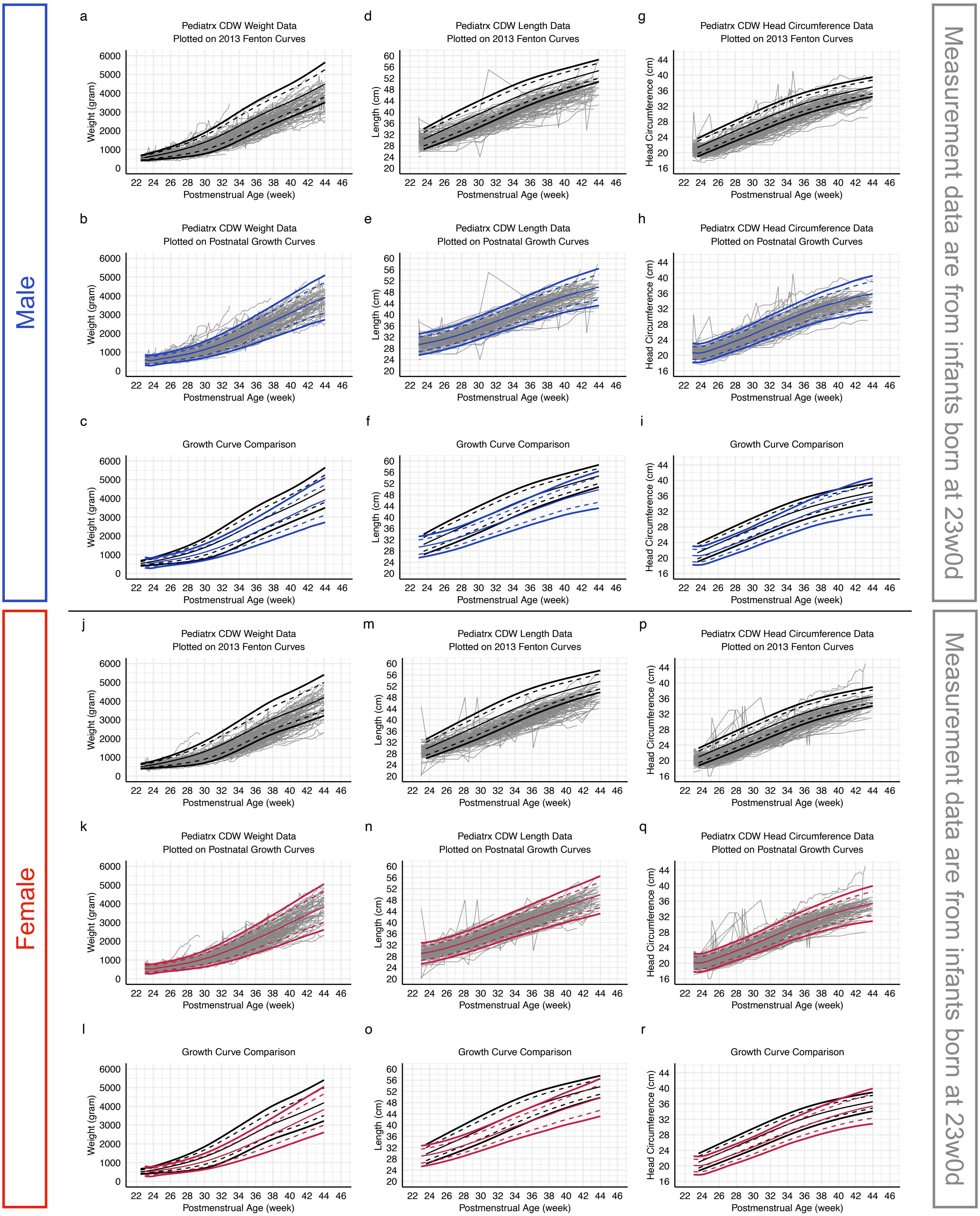

Figure S2. Postnatal and intrauterine growth comparison for the 24-week gestational age group. Comparing postnatal weight (a-c,j-l), length (d-f,m-o), and head circumference (g-i,p-r) models for male (a-i) and female (j-r) infants to the 2013 Fenton growth charts.

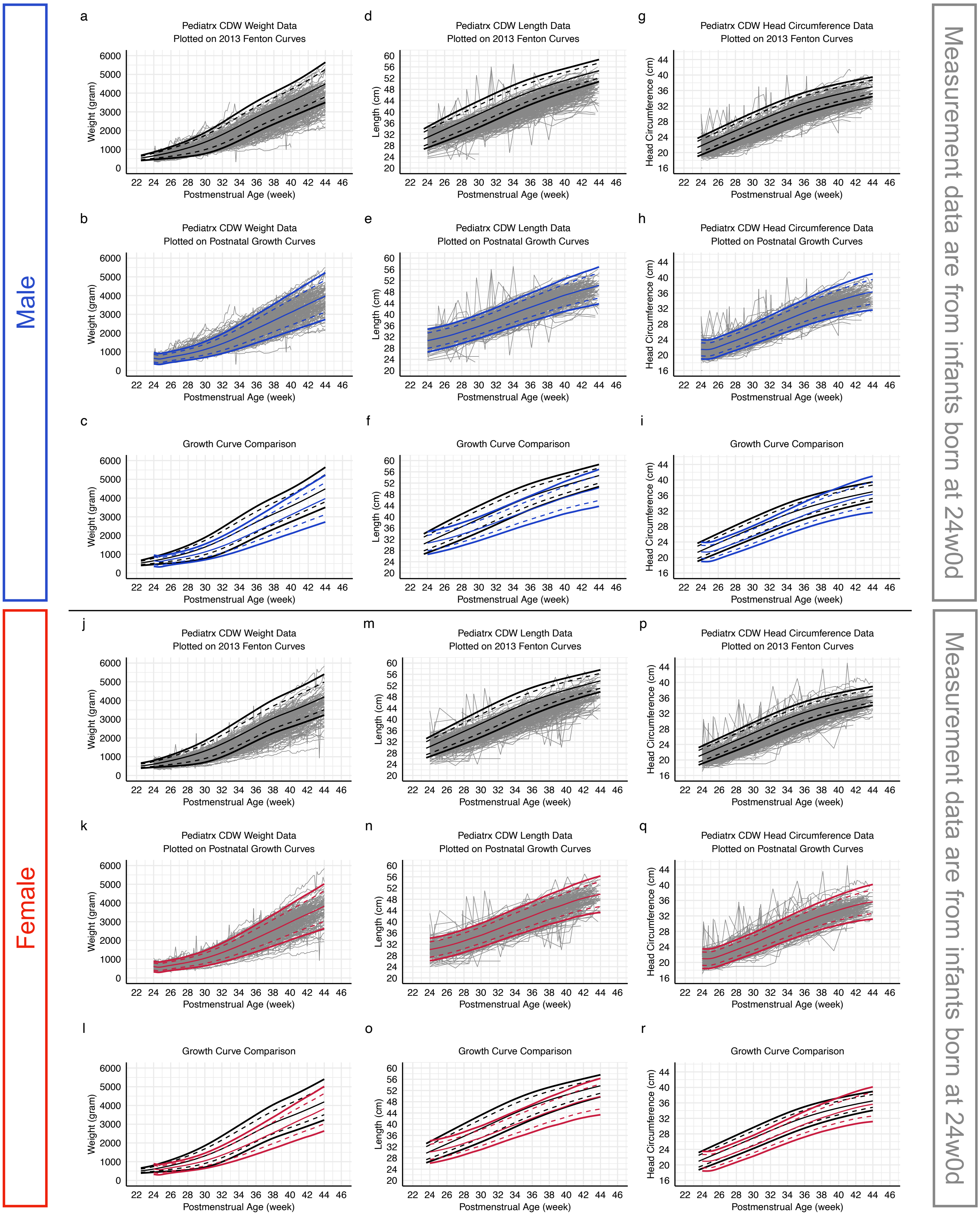

Figure S3. Postnatal and intrauterine growth comparison for the 25-week gestational age group. Comparing postnatal weight (a-c,j-l), length (d-f,m-o), and head circumference (g-i,p-r) models for male (a-i) and female (j-r) infants to the 2013 Fenton growth charts.

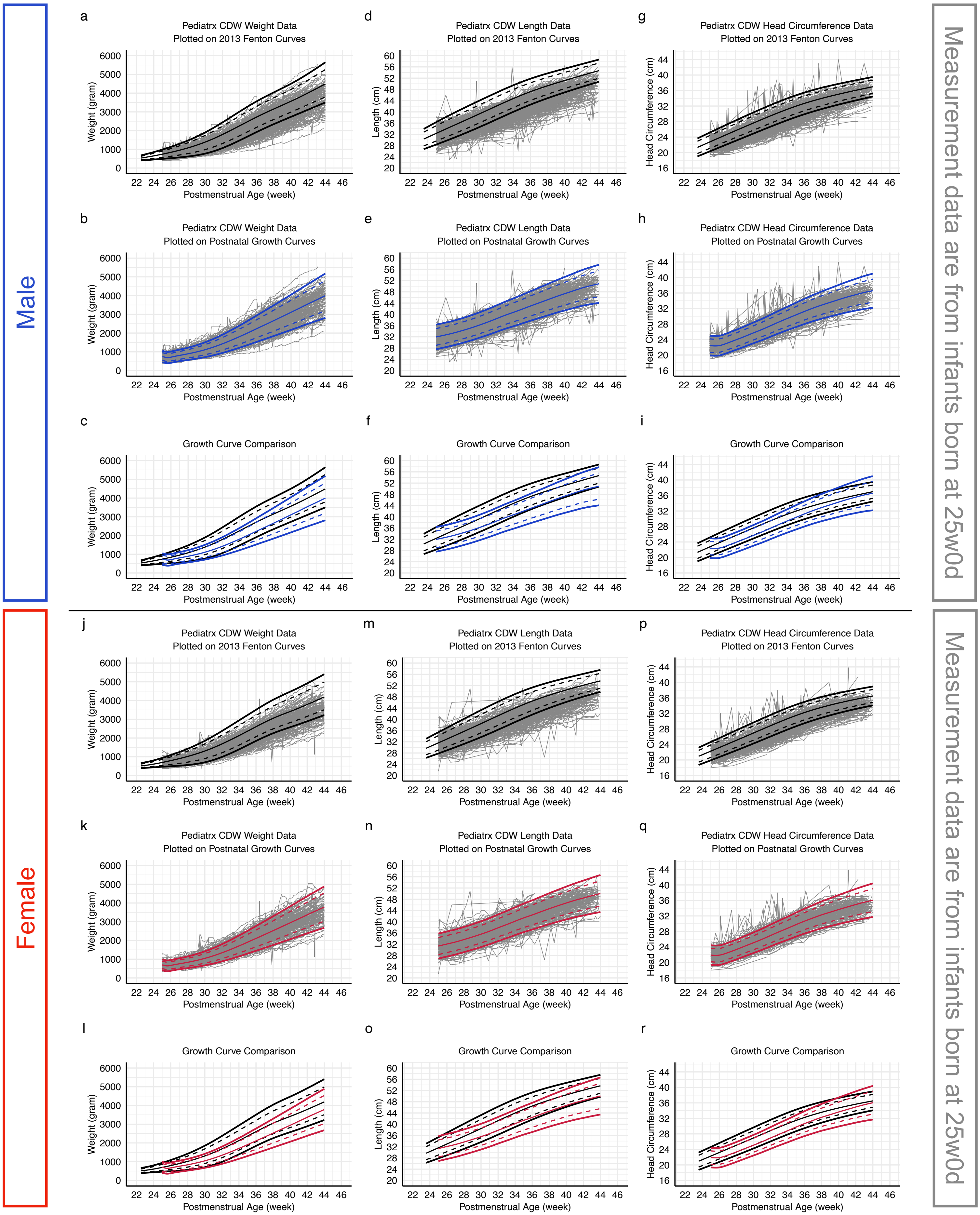

Figure S4. Postnatal and intrauterine growth comparison for the 26-week gestational age group. Comparing postnatal weight (a-c,j-l), length (d-f,m-o), and head circumference (g-i,p-r) models for male (a-i) and female (j-r) infants to the 2013 Fenton growth charts.

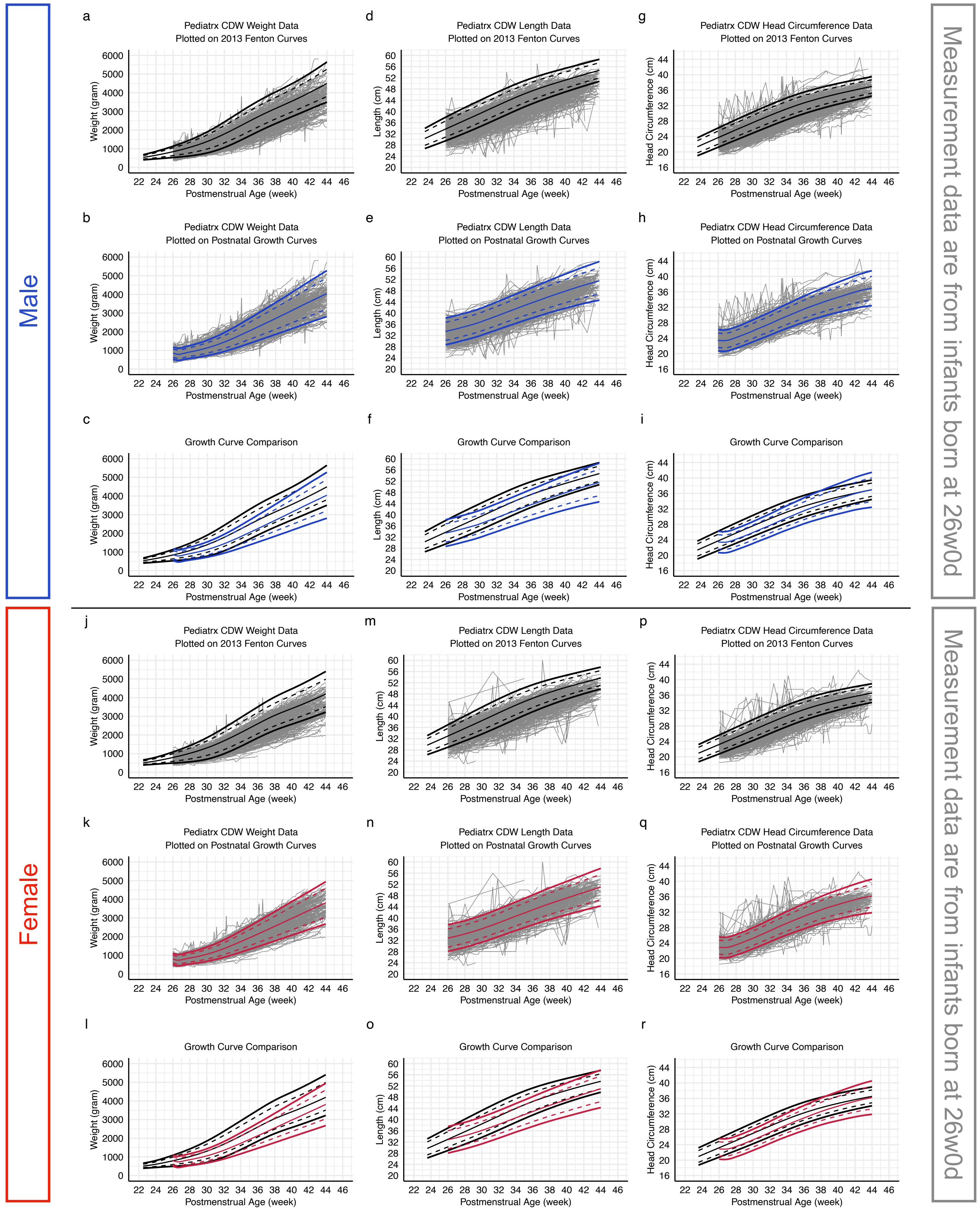

Figure S5. Postnatal and intrauterine growth comparison for the 27-week gestational age group. Comparing postnatal weight (a-c,j-l), length (d-f,m-o), and head circumference (g-i,p-r) models for male (a-i) and female (j-r) infants to the 2013 Fenton growth charts.

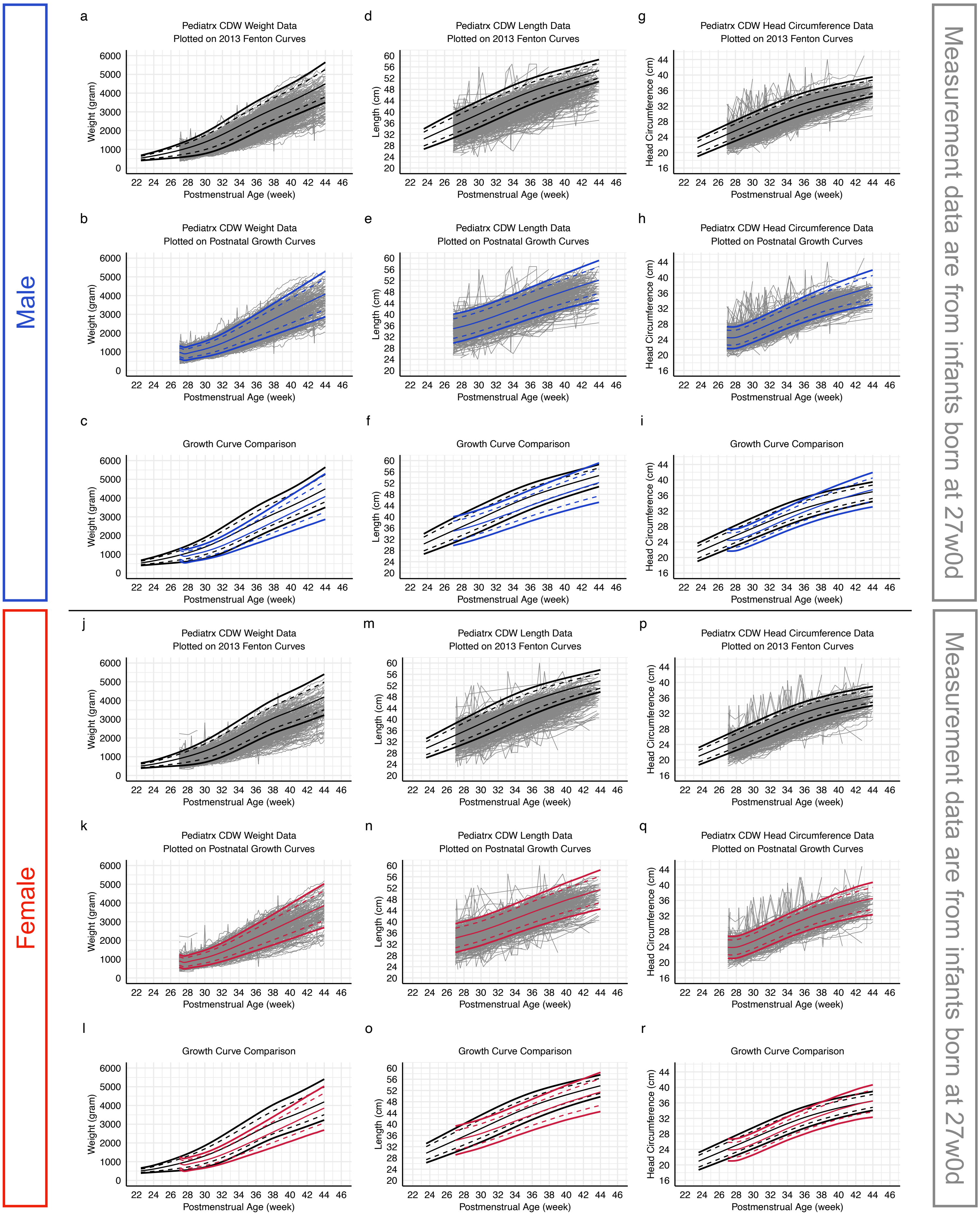

Figure S6. Postnatal and intrauterine growth comparison for the 28-week gestational age group. Comparing postnatal weight (a-c,j-l), length (d-f,m-o), and head circumference (g-i,p-r) models for male (a-i) and female (j-r) infants to the 2013 Fenton growth charts.

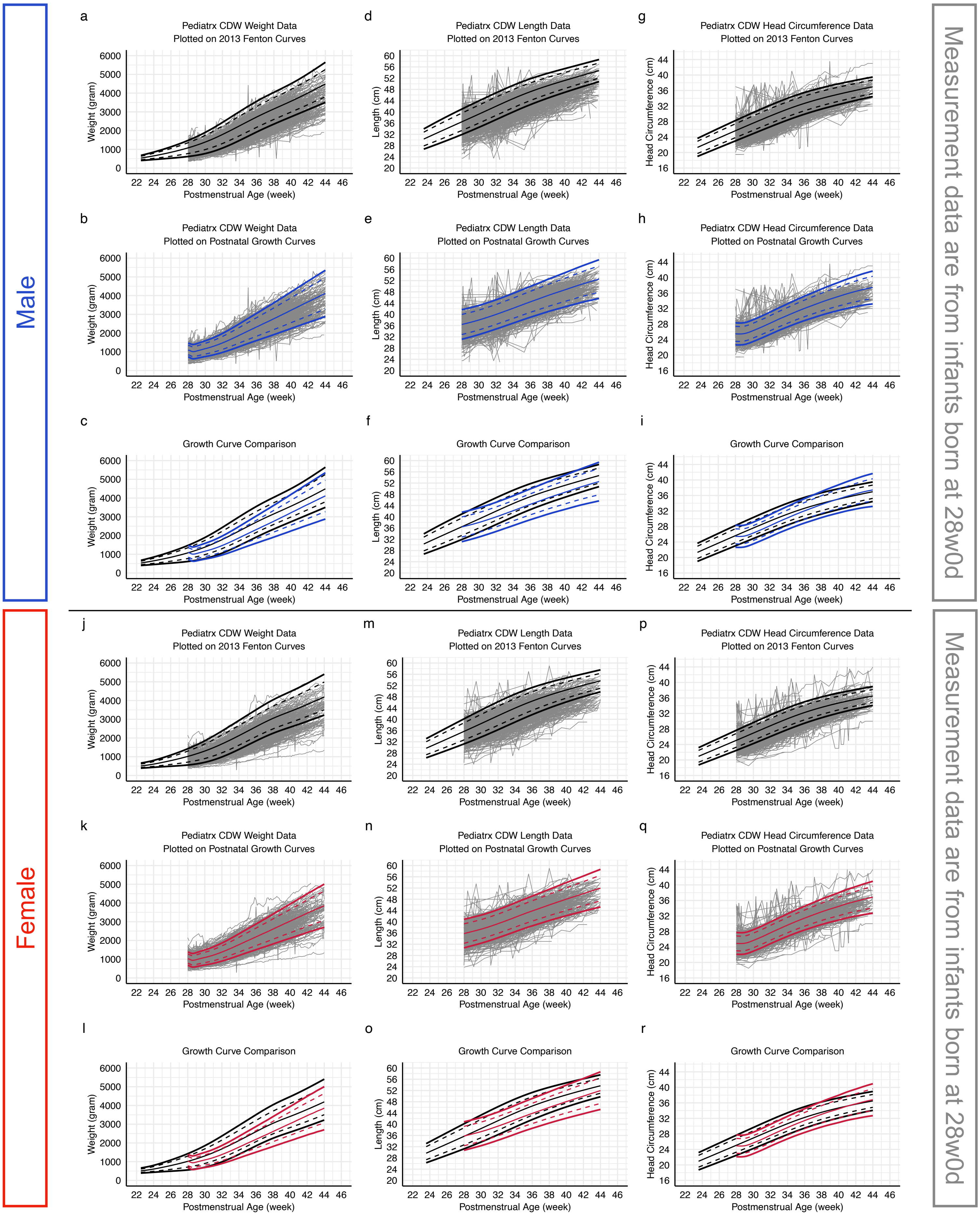

Figure S7. Postnatal and intrauterine growth comparison for the 29-week gestational age group. Comparing postnatal weight (a-c,j-l), length (d-f,m-o), and head circumference (g-i,p-r) models for male (a-i) and female (j-r) infants to the 2013 Fenton growth charts.

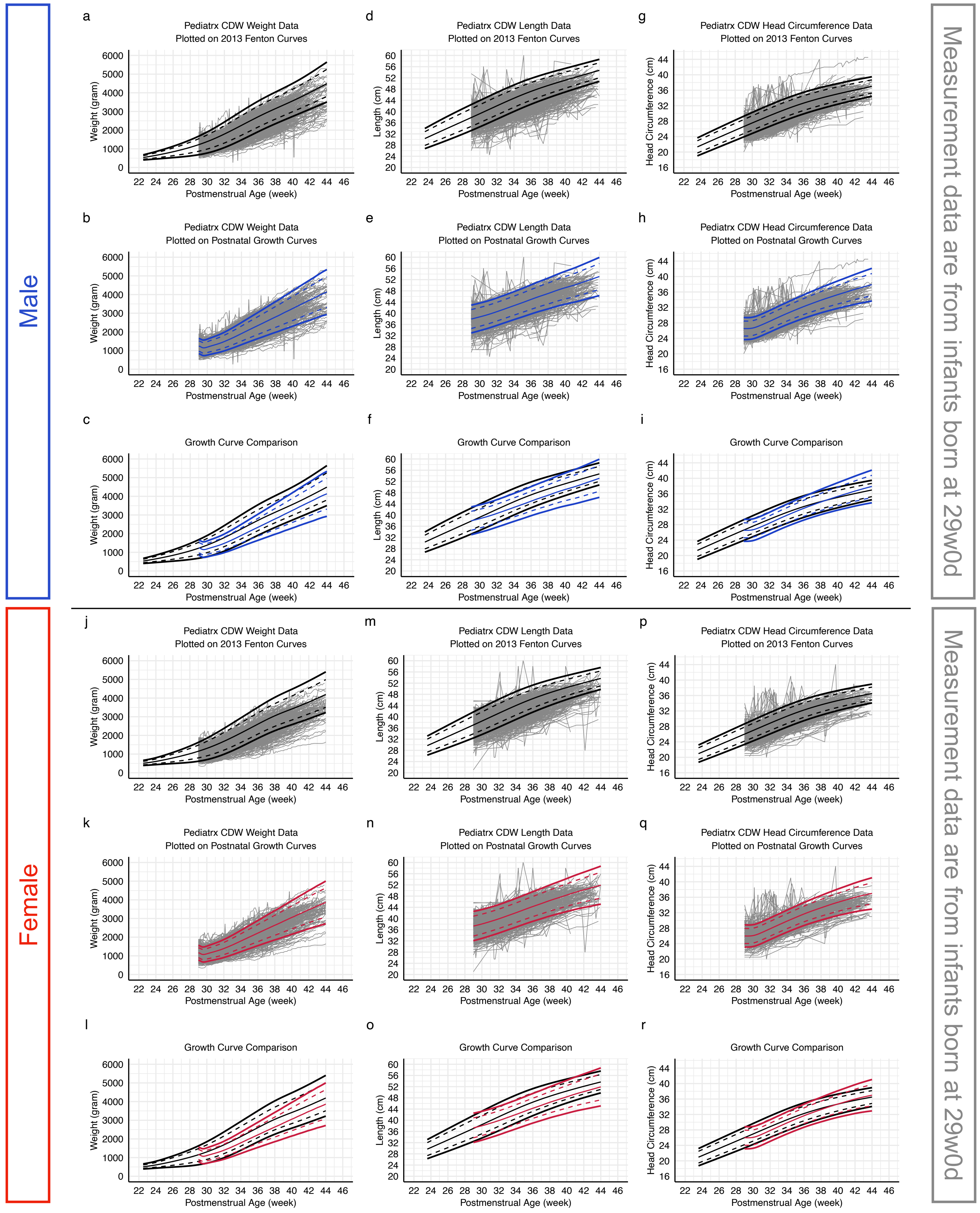

Figure S8. Postnatal and intrauterine growth comparison for the 30-week gestational age group. Comparing postnatal weight (a-c,j-l), length (d-f,m-o), and head circumference (g-i,p-r) models for male (a-i) and female (j-r) infants to the 2013 Fenton growth charts.

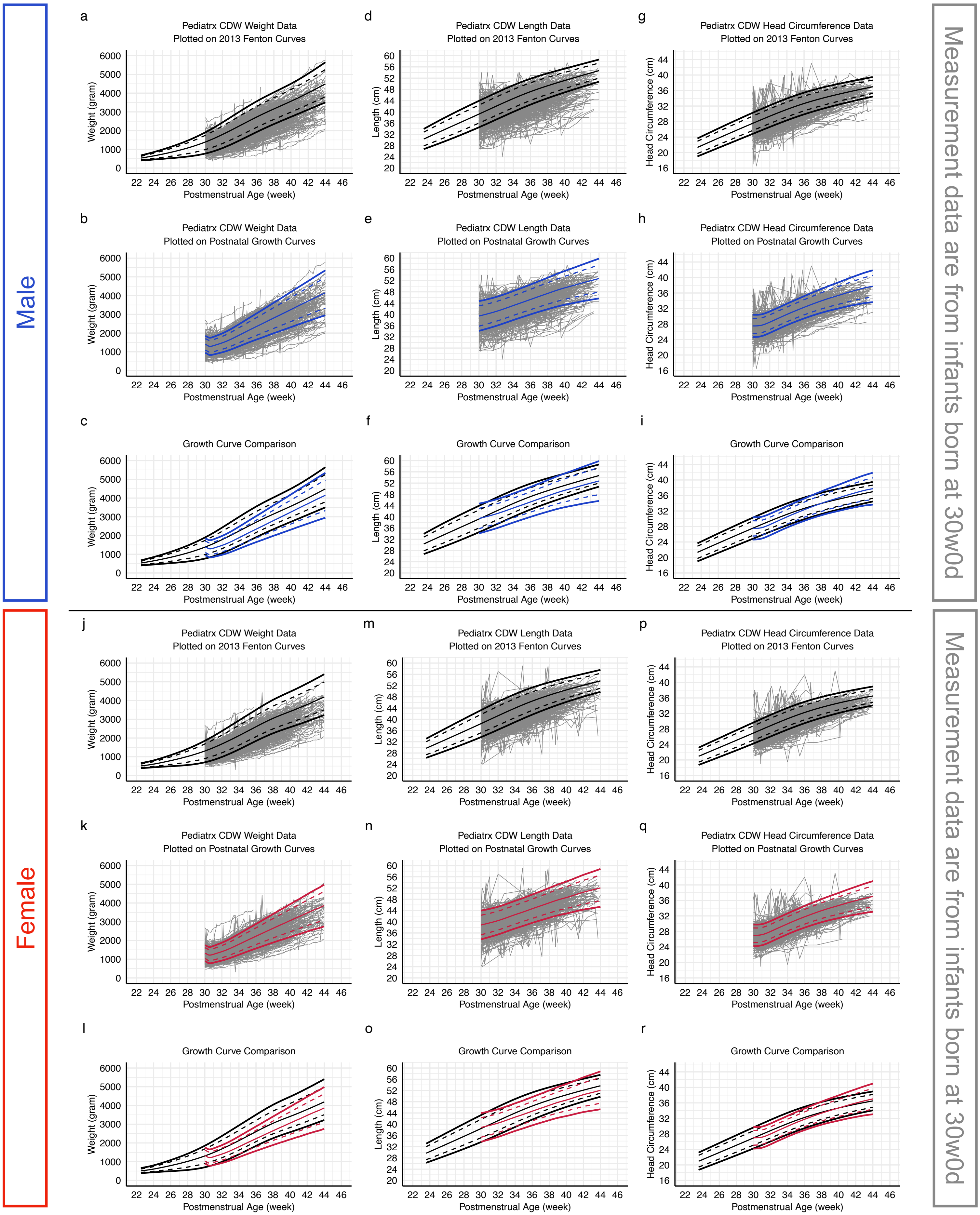

Figure S9. Marginal mean absolute error (MAE) and root-mean-square error (RMSE) comparison between the training and the validation datasets. The trained models for weight (a,b), length (c,d), and head circumference (e,f) developed using measurement values from randomly selected 70% of infants for each gestational age and sex (male: a,c,e; female: b,d,f) group (the training dataset) was applied to make predictions on the measurement values from the remaining 30% of infants (the validation dataset) as well as the training dataset. Marginal MAE and RMSE were calculated for comparison to examine overfitting. Open and closed circles represent MAE or RMSE calculated using the training and the validation datasets, respectively.

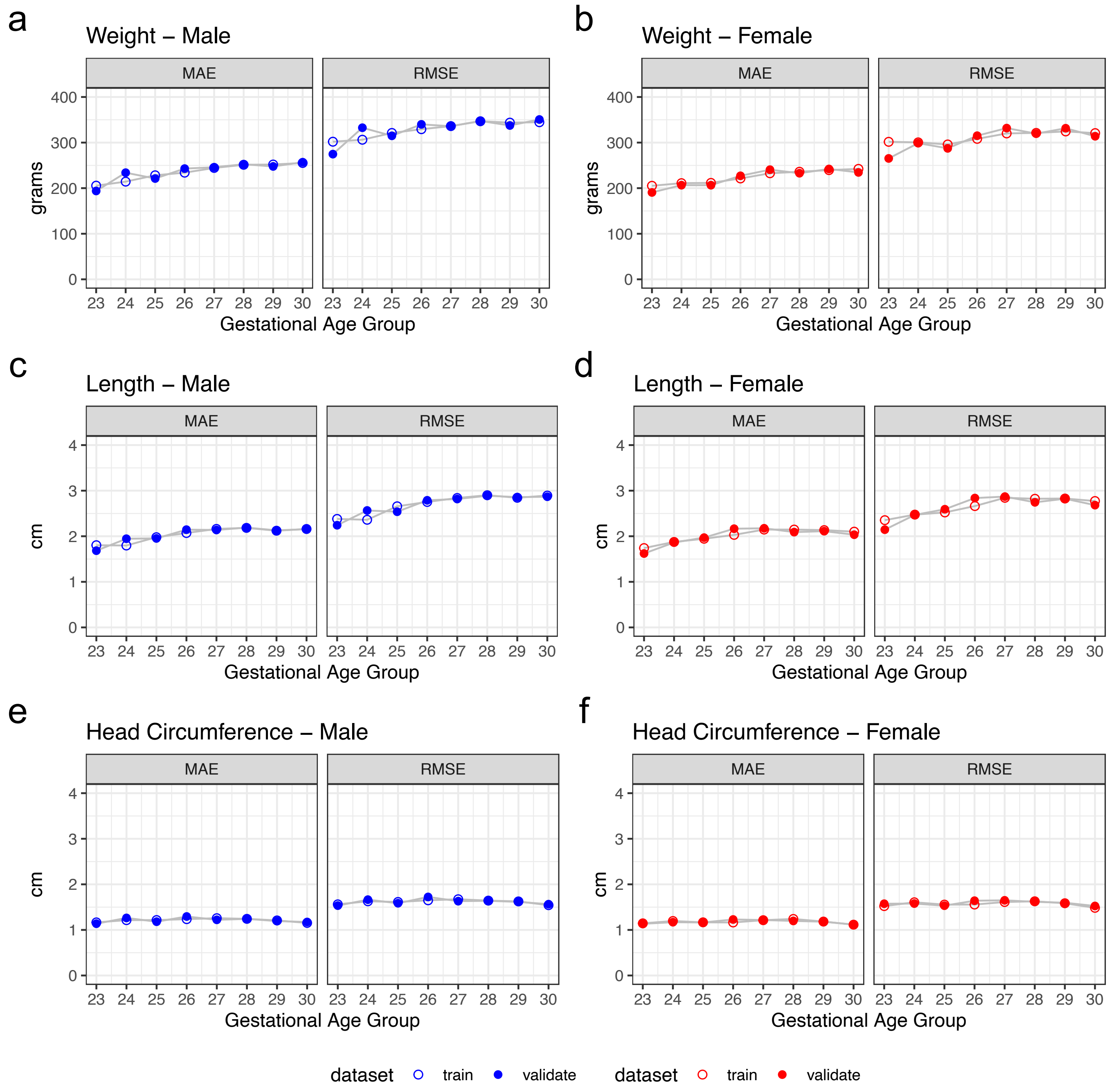

Figure S10. R-squared comparison between the training and the validation datasets. R-Squared was calculated for each gestational age and sex group (male: a,c,e; female: b,d,f) using both training and validation dataset for comparison to assess overfitting of the trained models for weight (a,b), length (c,d), and head circumference (e,f). Open and closed circles represent R-squared calculated using the training and the validation datasets, respectively.

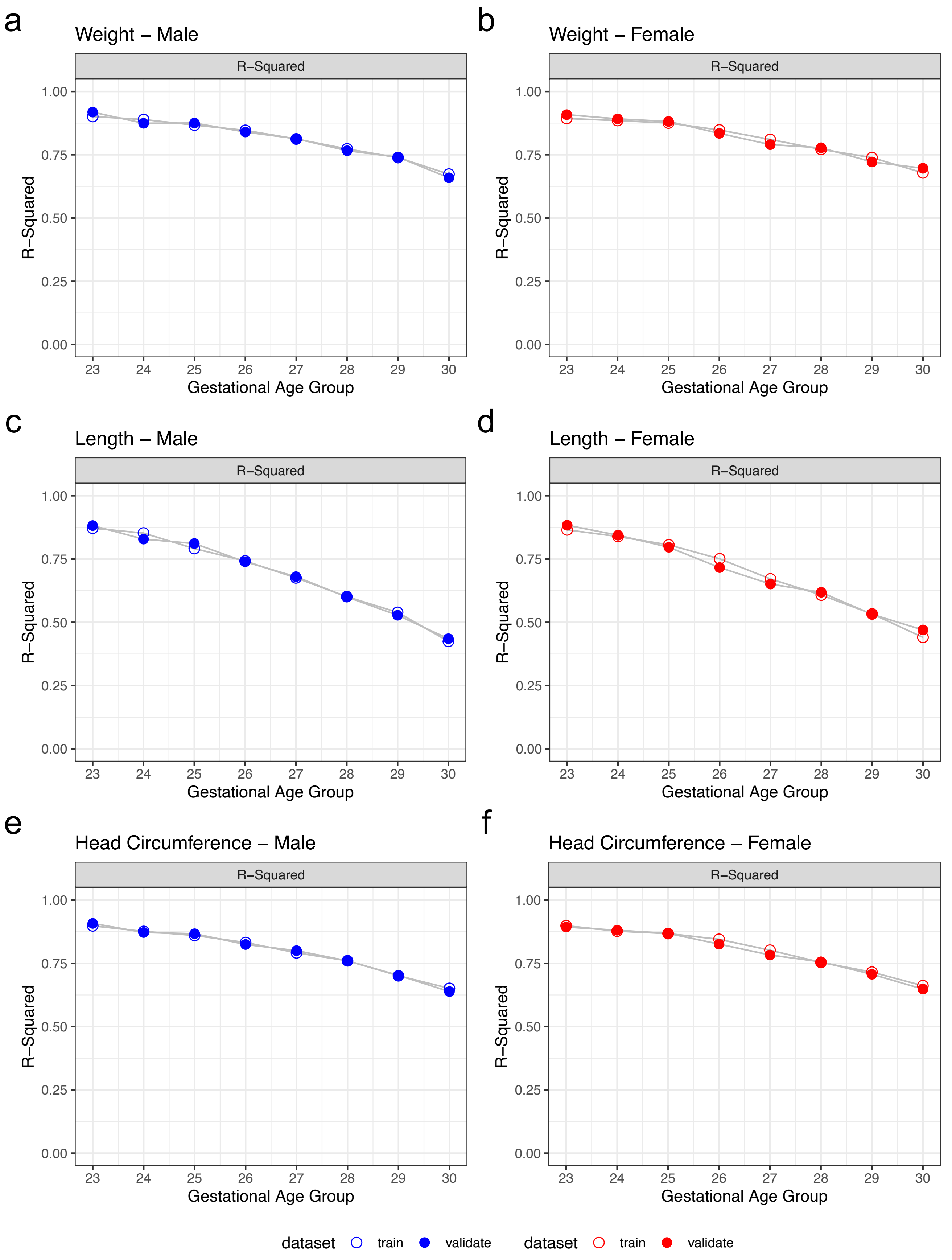

Figure S11. Postnatal and intrauterine growth rate comparison for the 23-week gestational age group. Comparing postnatal weight (a,d), length (b,e), and head circumference (c,f) growth rates for male (a-c) and female (d-f) infants to the intrauterine growth rates

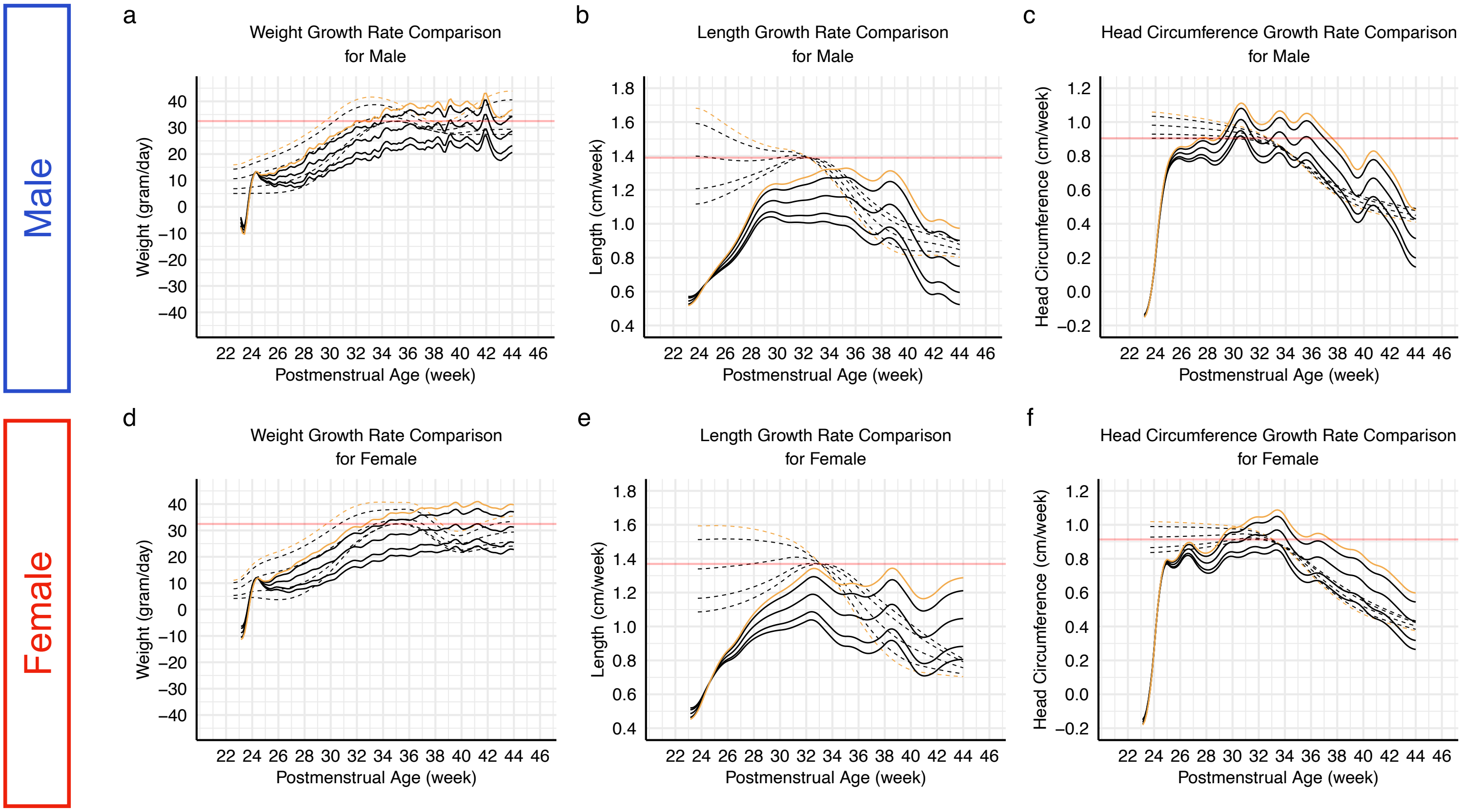

Figure S12. Postnatal and intrauterine growth rate comparison for the 24-week gestational age group. Comparing postnatal weight (a,d), length (b,e), and head circumference (c,f) growth rates for male (a-c) and female (d-f) infants to the intrauterine growth rates

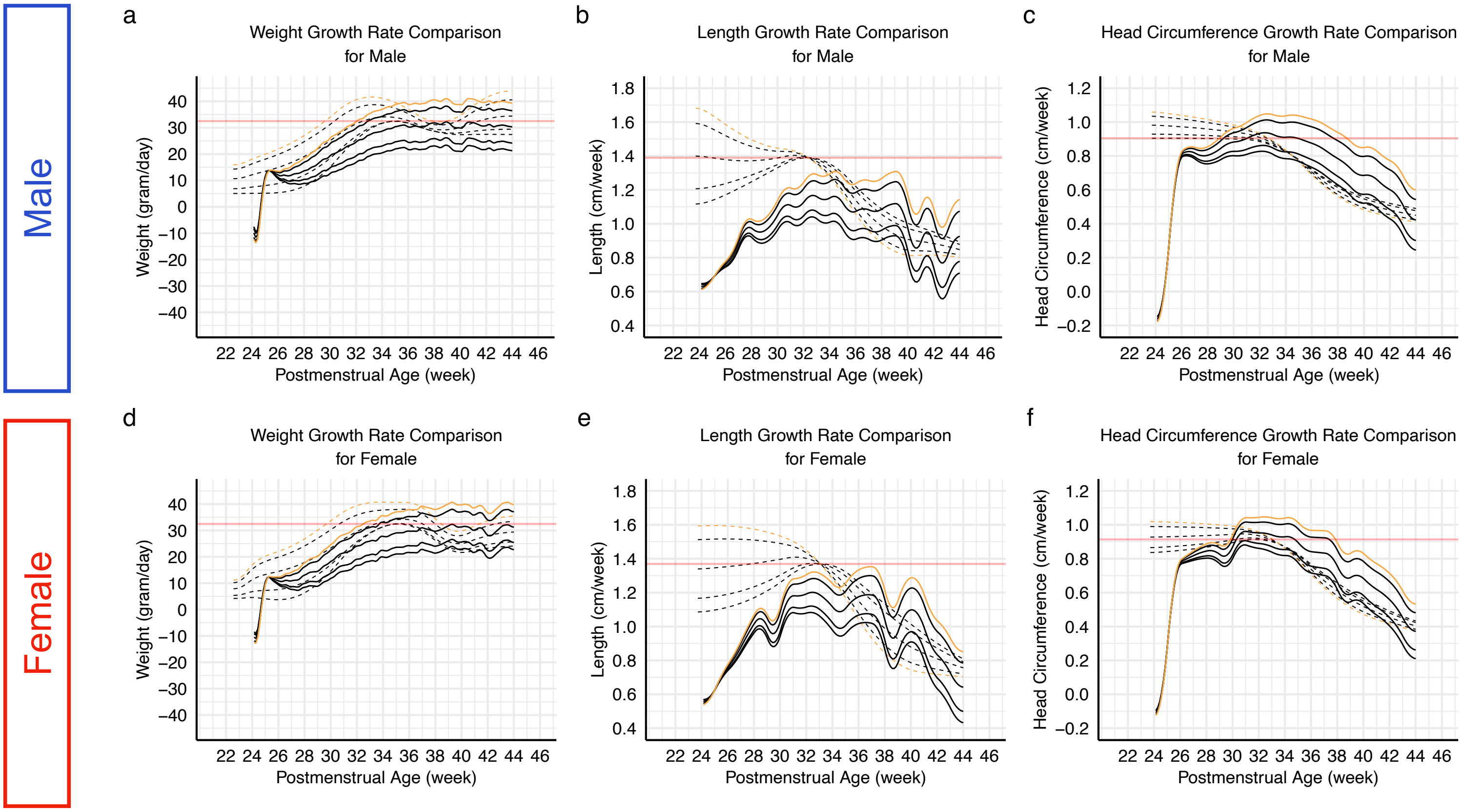

Figure S13. Postnatal and intrauterine growth rate comparison for the 25-week gestational age group. Comparing postnatal weight (a,d), length (b,e), and head circumference (c,f) growth rates for male (a-c) and female (d-f) infants to the intrauterine growth rates

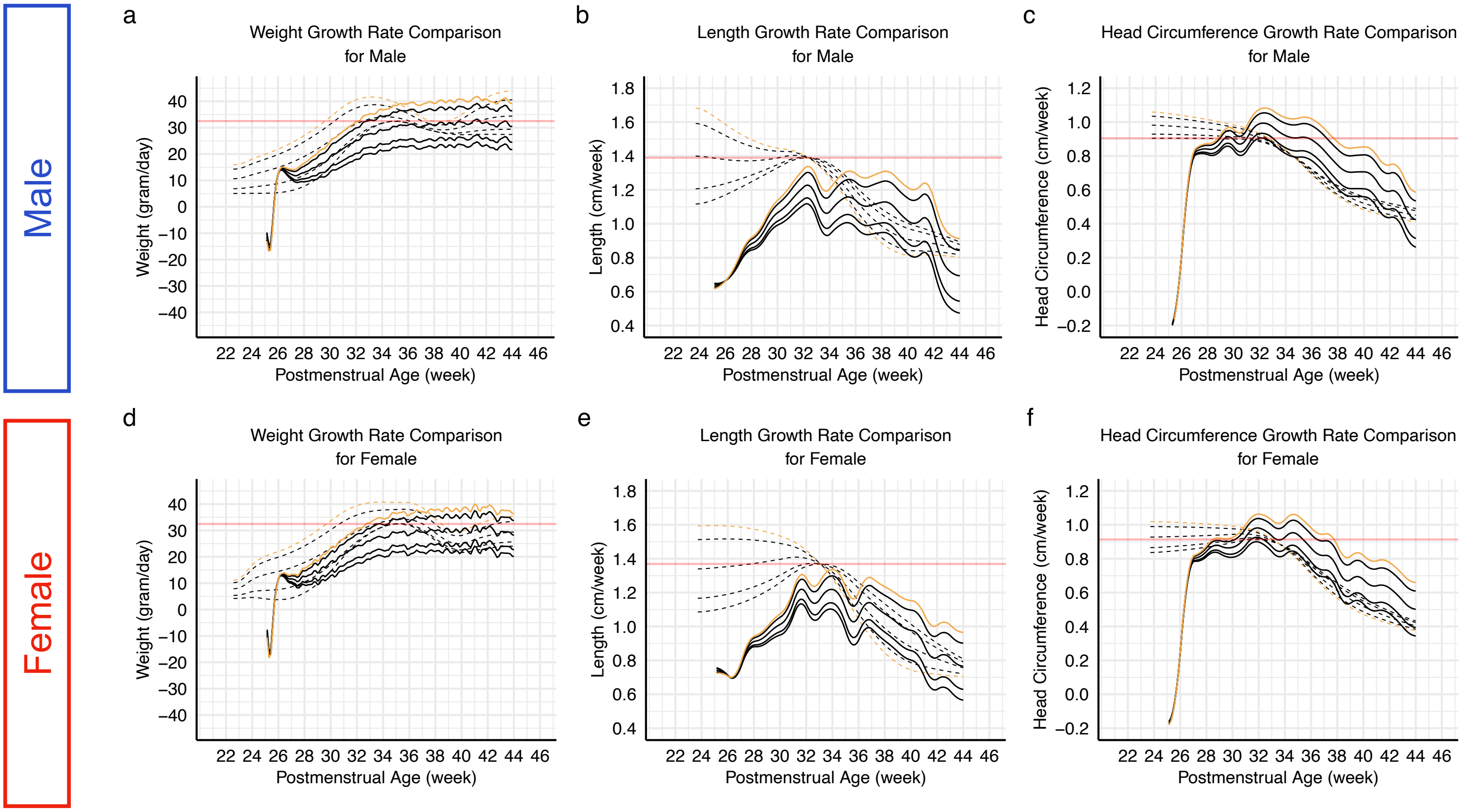

Figure S14. Postnatal and intrauterine growth rate comparison for the 26-week gestational age group. Comparing postnatal weight (a,d), length (b,e), and head circumference (c,f) growth rates for male (a-c) and female (d-f) infants to the intrauterine growth rates

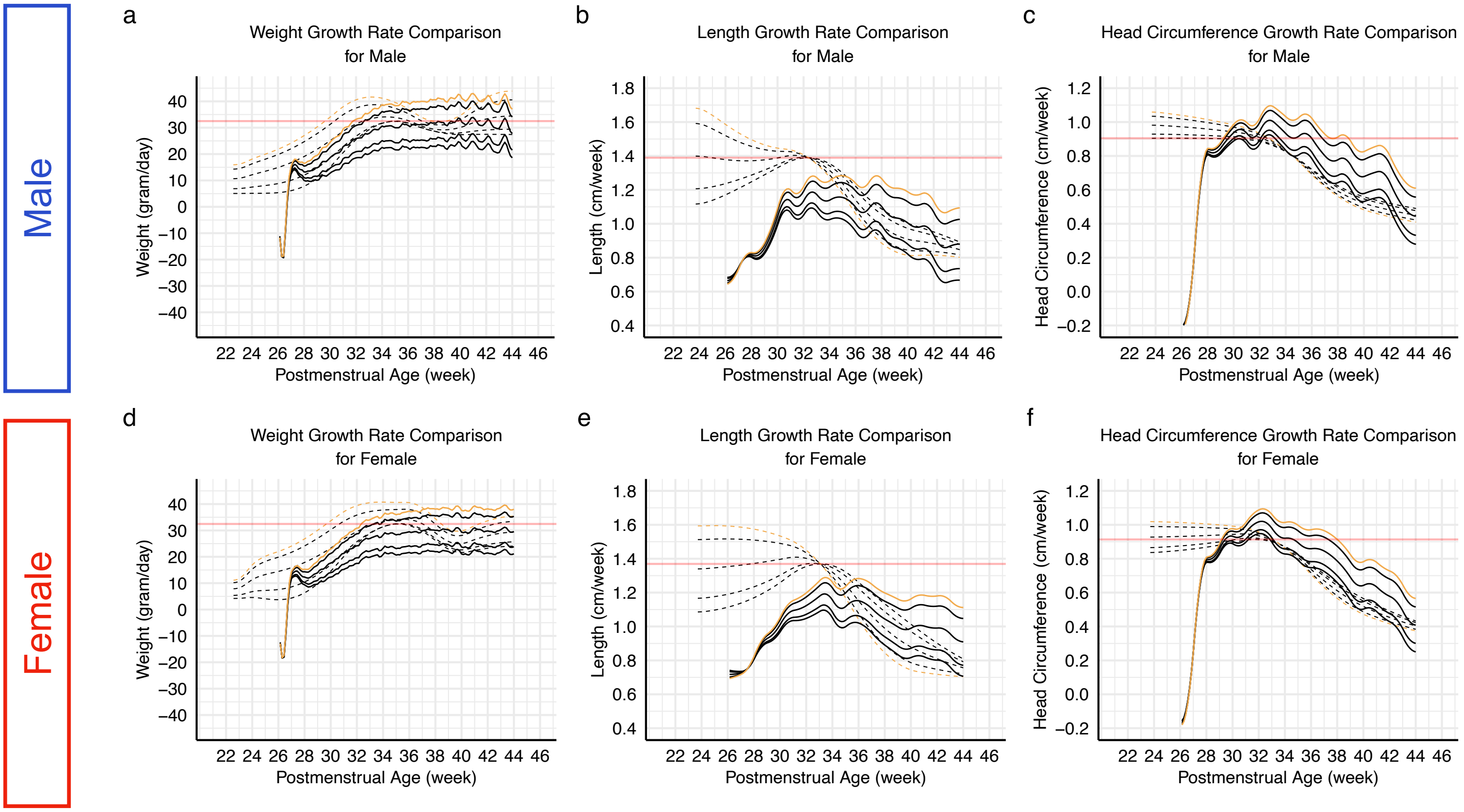

Figure S15. Postnatal and intrauterine growth rate comparison for the 27-week gestational age group. Comparing postnatal weight (a,d), length (b,e), and head circumference (c,f) growth rates for male (a-c) and female (d-f) infants to the intrauterine growth rates

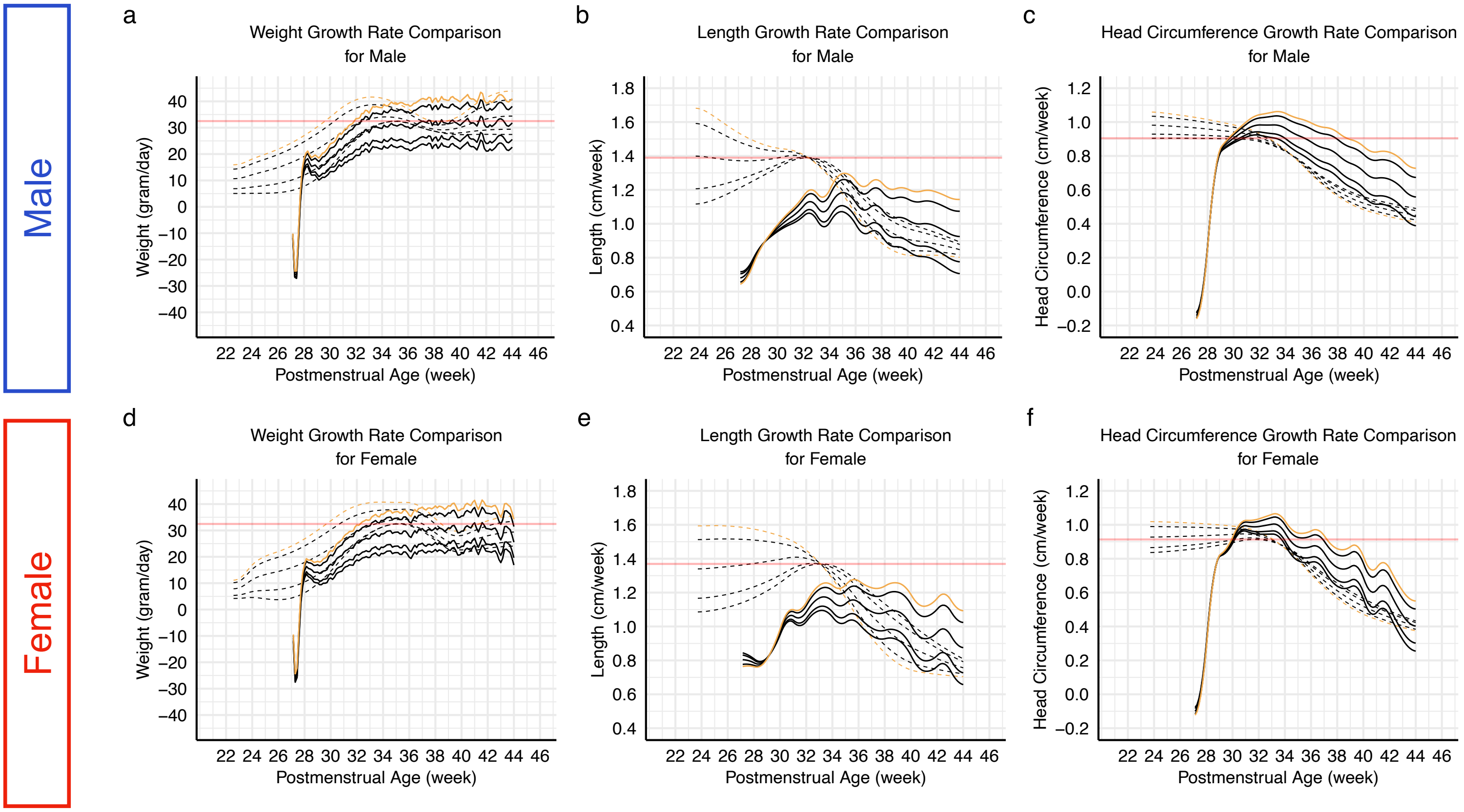

Figure S16. Postnatal and intrauterine growth rate comparison for the 28-week gestational age group. Comparing postnatal weight (a,d), length (b,e), and head circumference (c,f) growth rates for male (a-c) and female (d-f) infants to the intrauterine growth rates

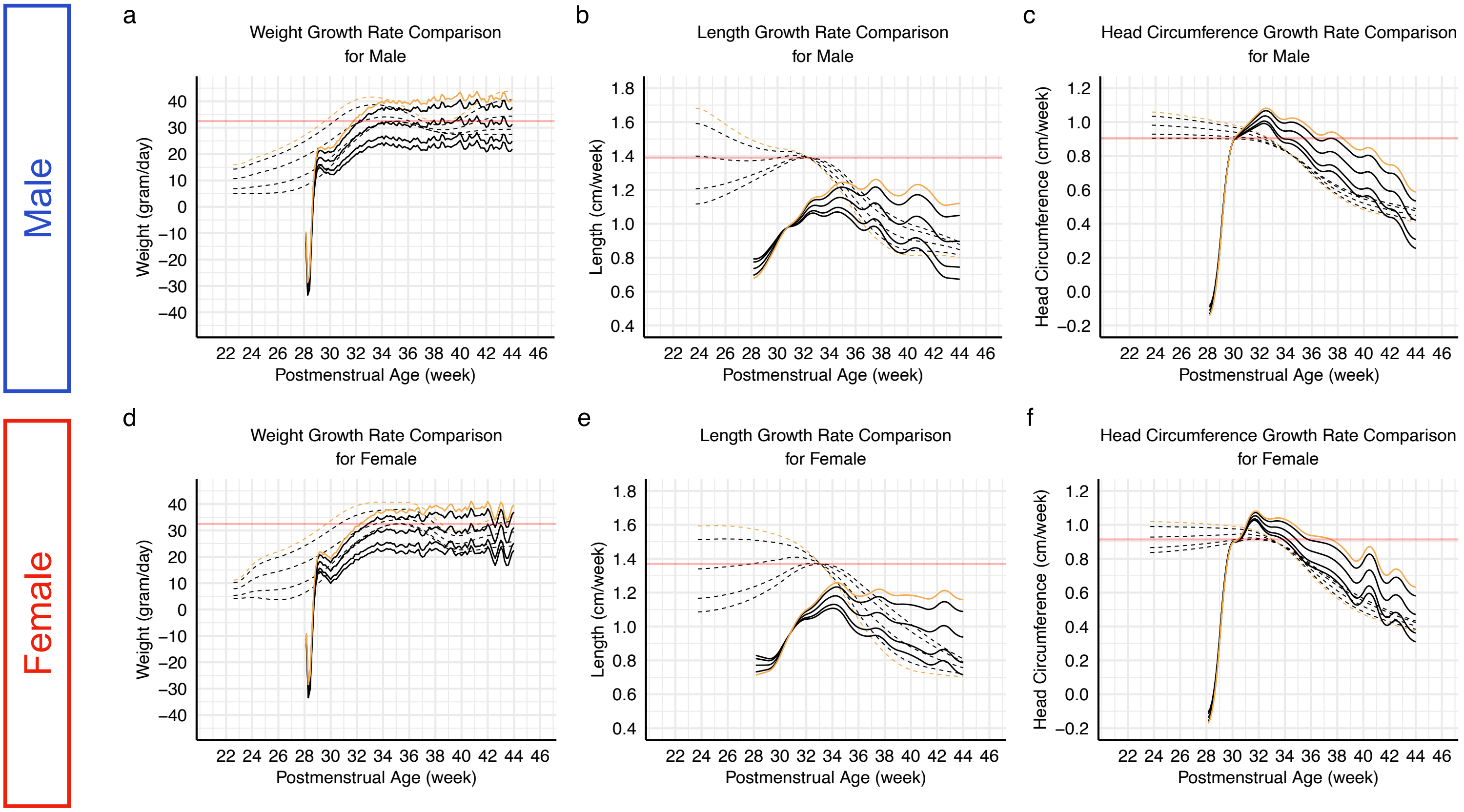

Figure S17. Postnatal and intrauterine growth rate comparison for the 29-week gestational age group. Comparing postnatal weight (a,d), length (b,e), and head circumference (c,f) growth rates for male (a-c) and female (d-f) infants to the intrauterine growth rates

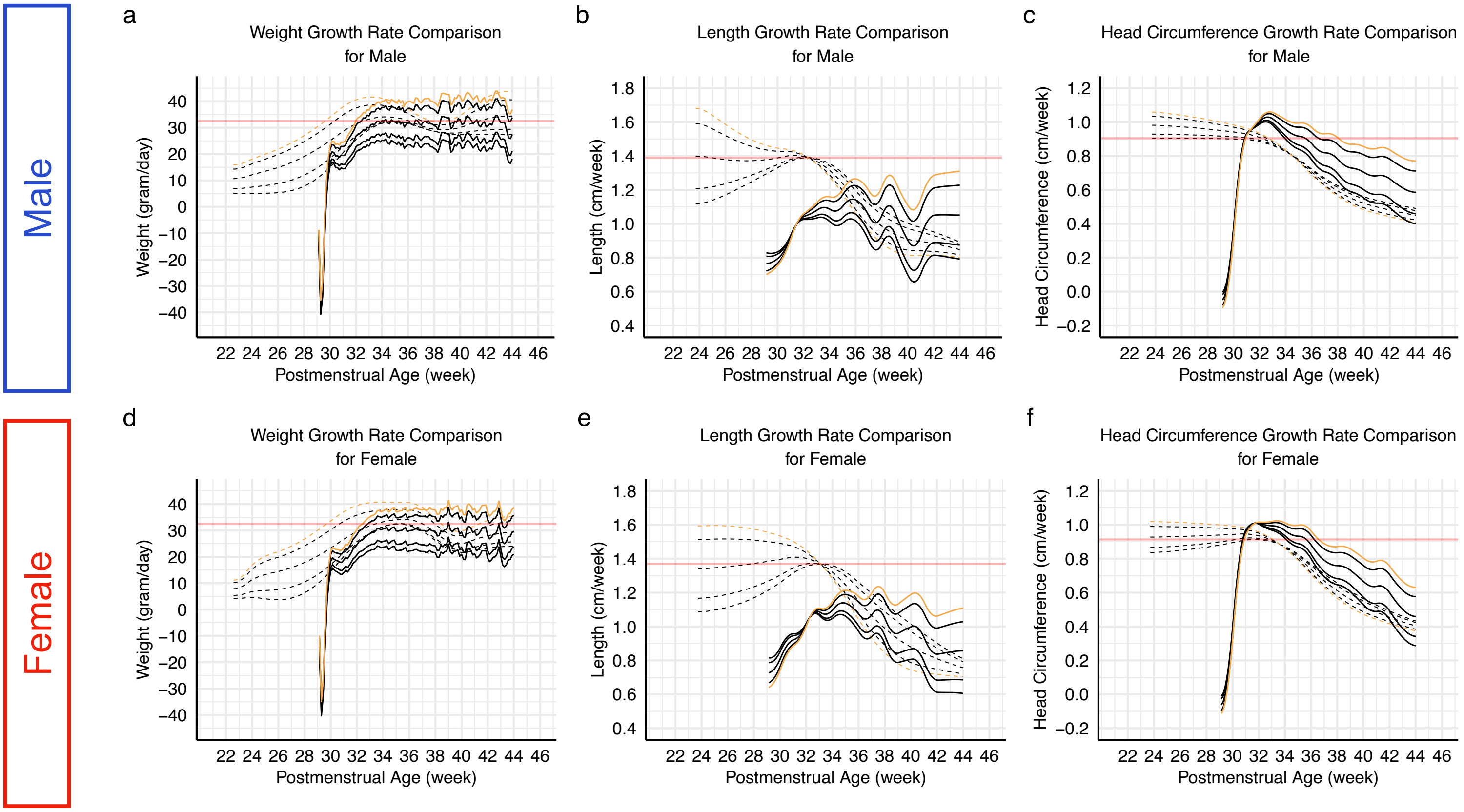

Figure S18. Postnatal and intrauterine growth rate comparison for the 30-week gestational age group. Comparing postnatal weight (a,d), length (b,e), and head circumference (c,f) growth rates for male (a-c) and female (d-f) infants to the intrauterine growth rates

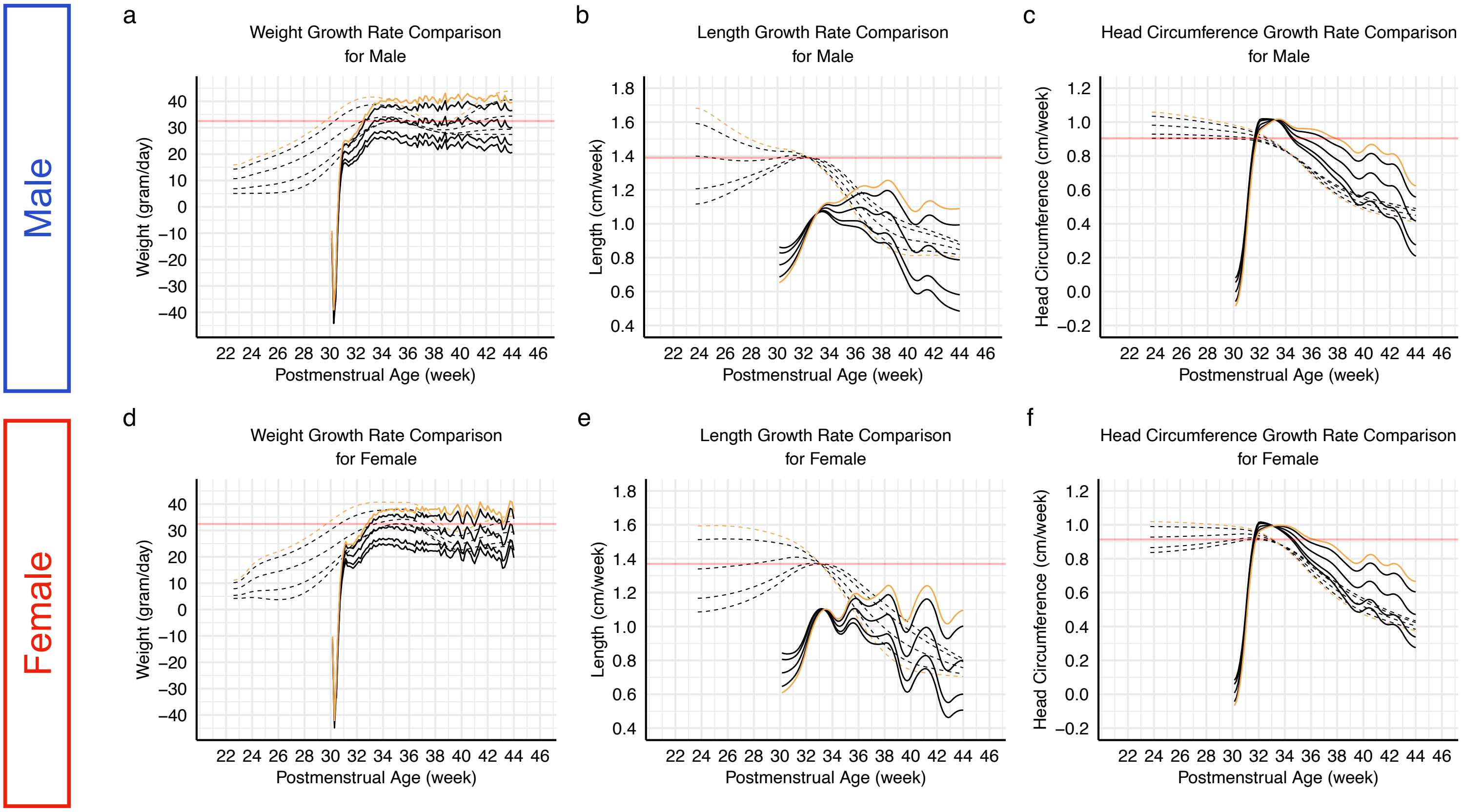

Figure S19. Estimated growth for the 3rd percentile. Growth trajectories (a,d) and growth rates during the weight acceleration (b,e) and the stable weight gain phases (c,f) for 23-30-week gestational age groups.

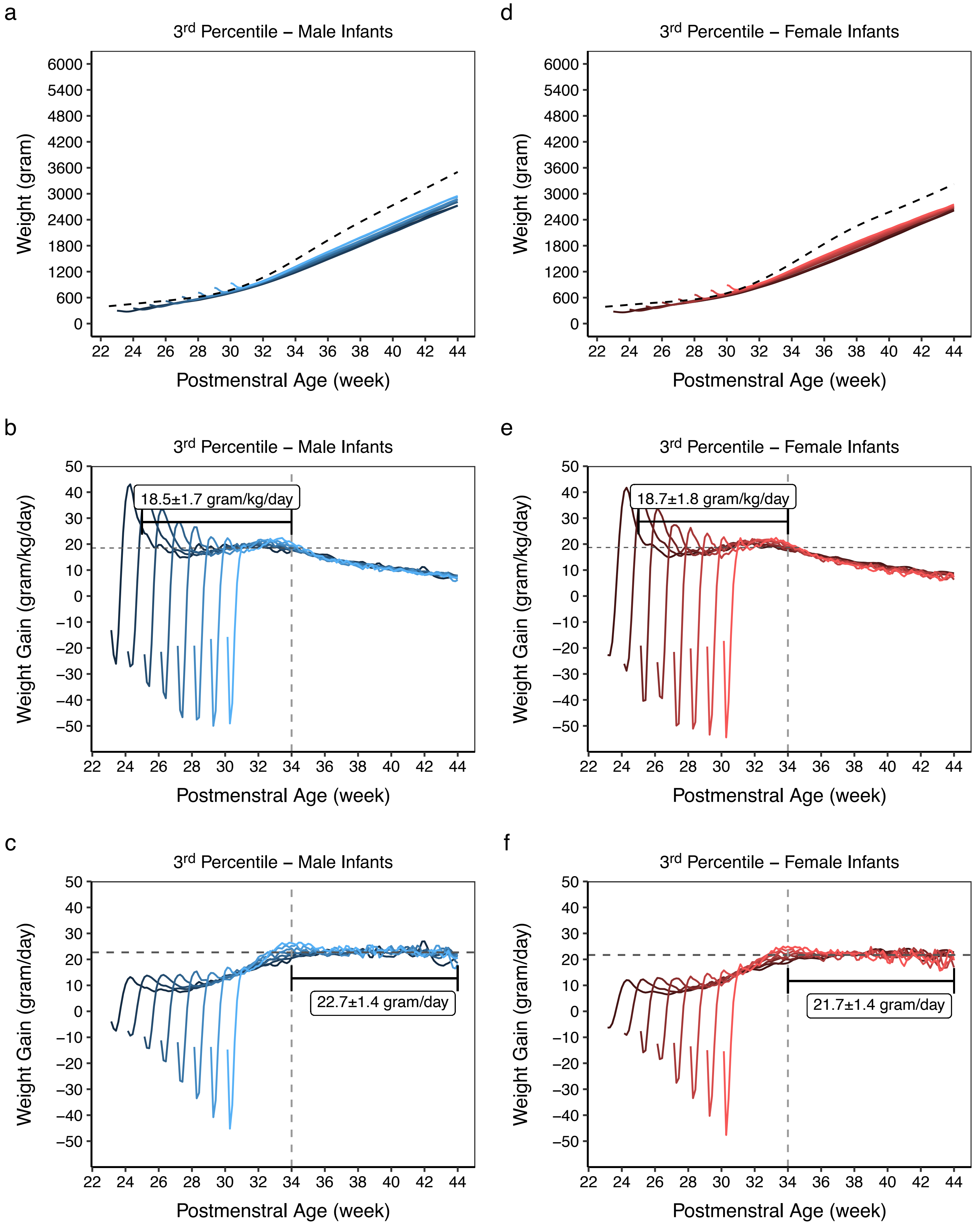

Figure S20. Estimated growth for the 10th percentile. Growth trajectories (a,d) and growth rates during the weight acceleration (b,e) and the stable weight gain phases (c,f) for 23-30-week gestational age groups.

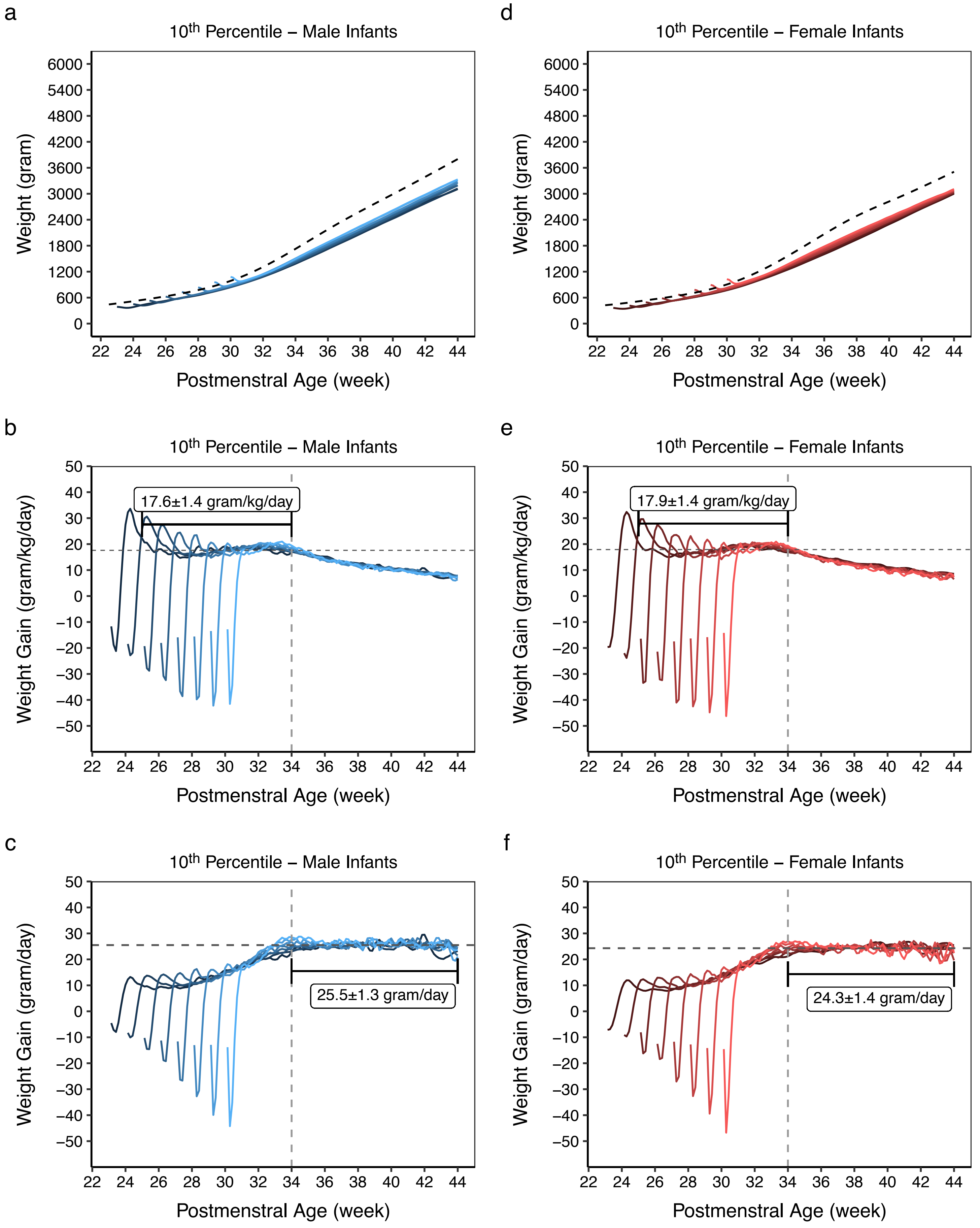

Figure S21. Estimated growth for the 25th percentile. Growth trajectories (a,d) and growth rates during the weight acceleration (b,e) and the stable weight gain phases (c,f) for 23-30-week gestational age groups.

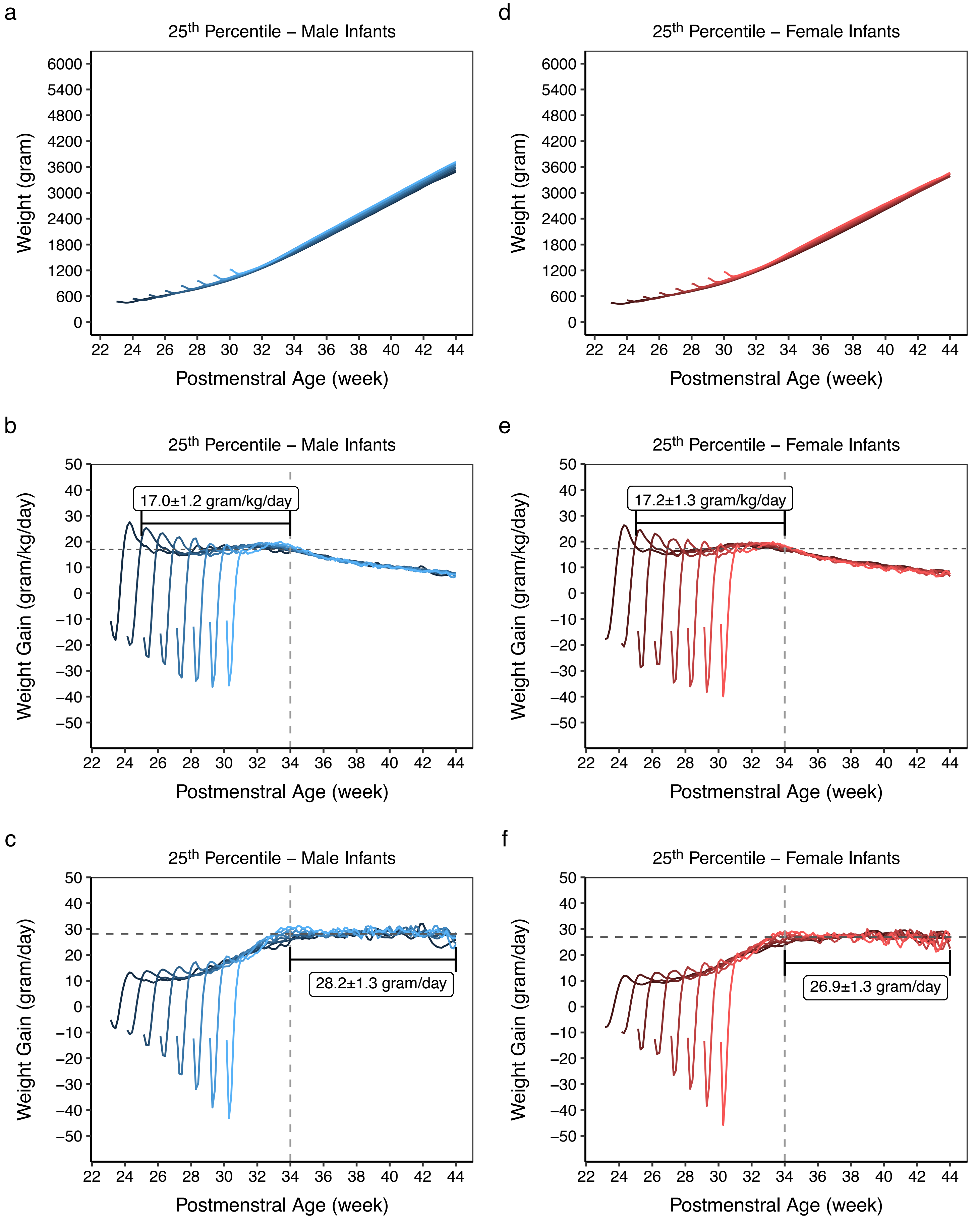

Figure S22. Estimated growth for the 50th percentile. Growth trajectories (a,d) and growth rates during the weight acceleration (b,e) and the stable weight gain phases (c,f) for 23-30-week gestational age groups.

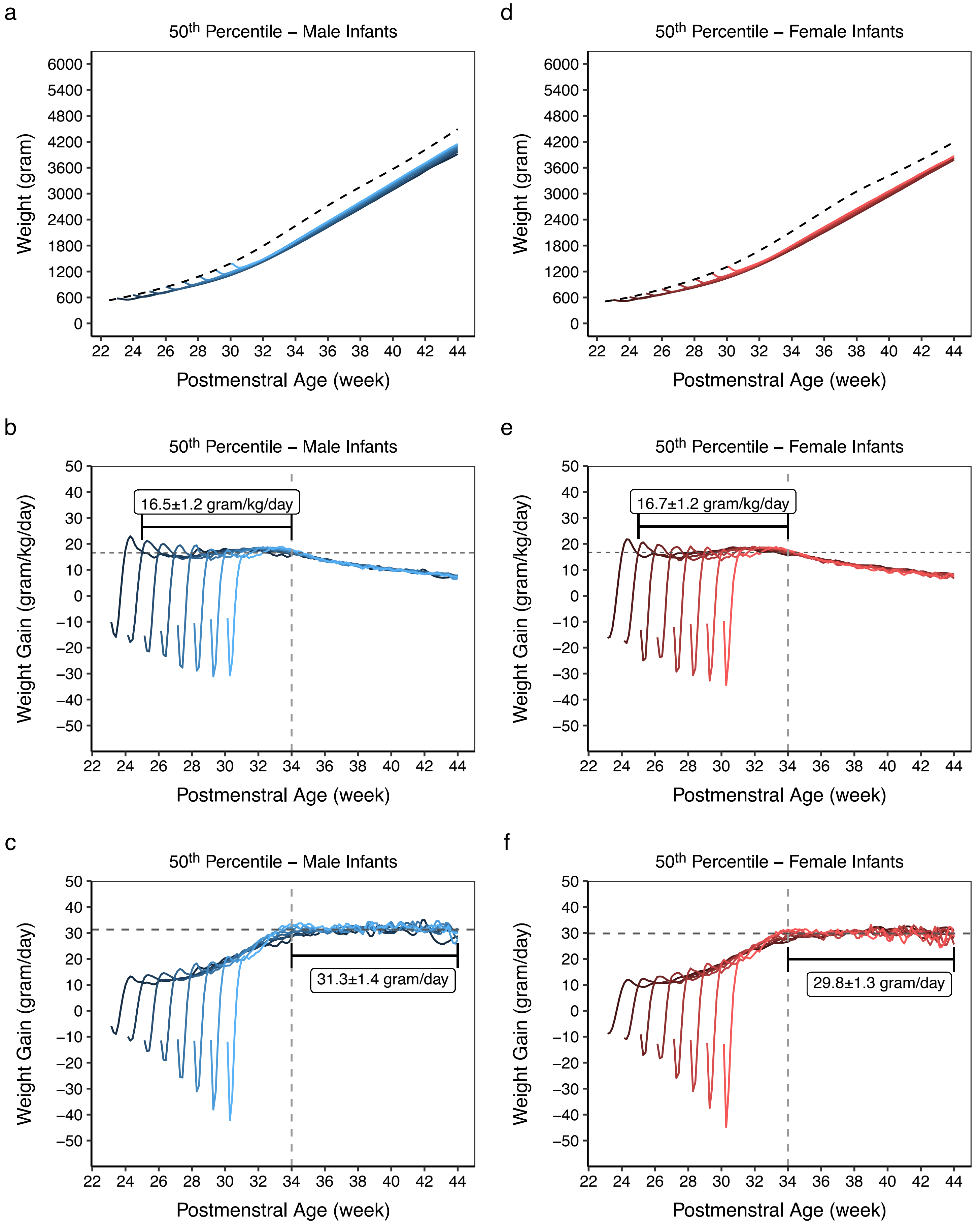

Figure S23. Estimated growth for the 75th percentile. Growth trajectories (a,d) and growth rates during the weight acceleration (b,e) and the stable weight gain phases (c,f) for 23-30-week gestational age groups.

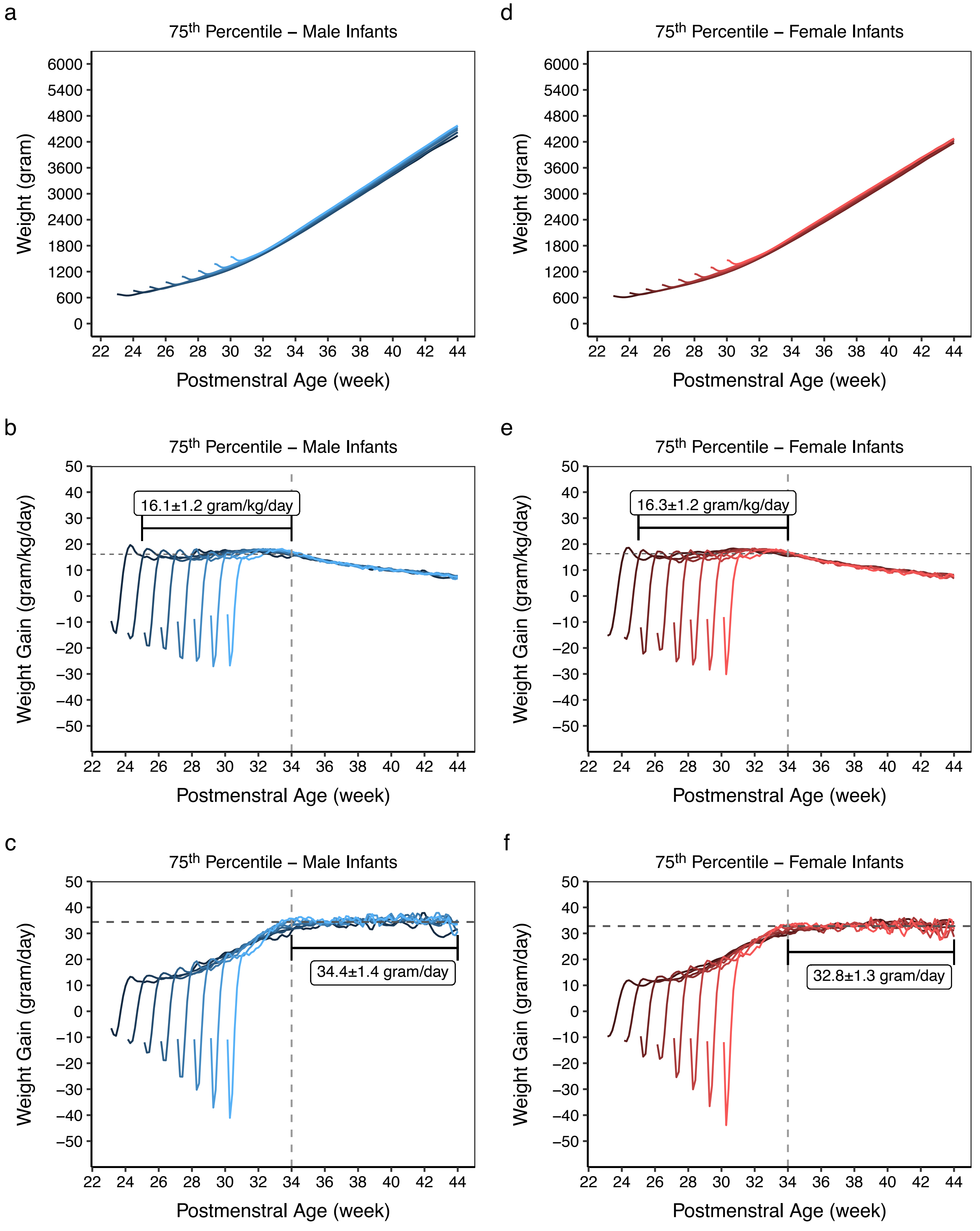

Figure S24. Estimated growth for the 90th percentile. Growth trajectories (a,d) and growth rates during the weight acceleration (b,e) and the stable weight gain phases (c,f) for 23-30-week gestational age groups.

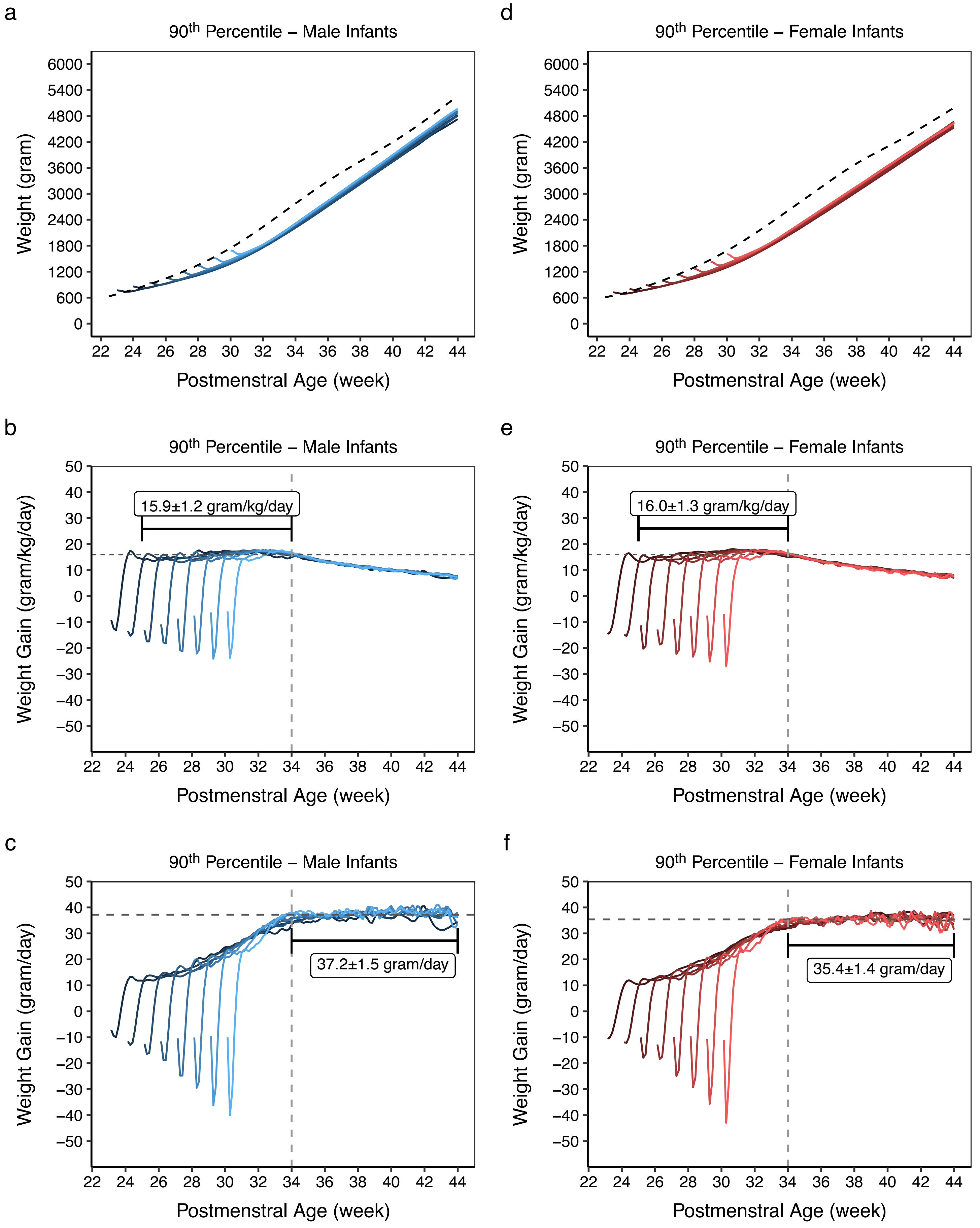

Figure S25. Estimated growth for the 97th percentile. Growth trajectories (a,d) and growth rates during the weight acceleration (b,e) and the stable weight gain phases (c,f) for 23-30-week gestational age groups.

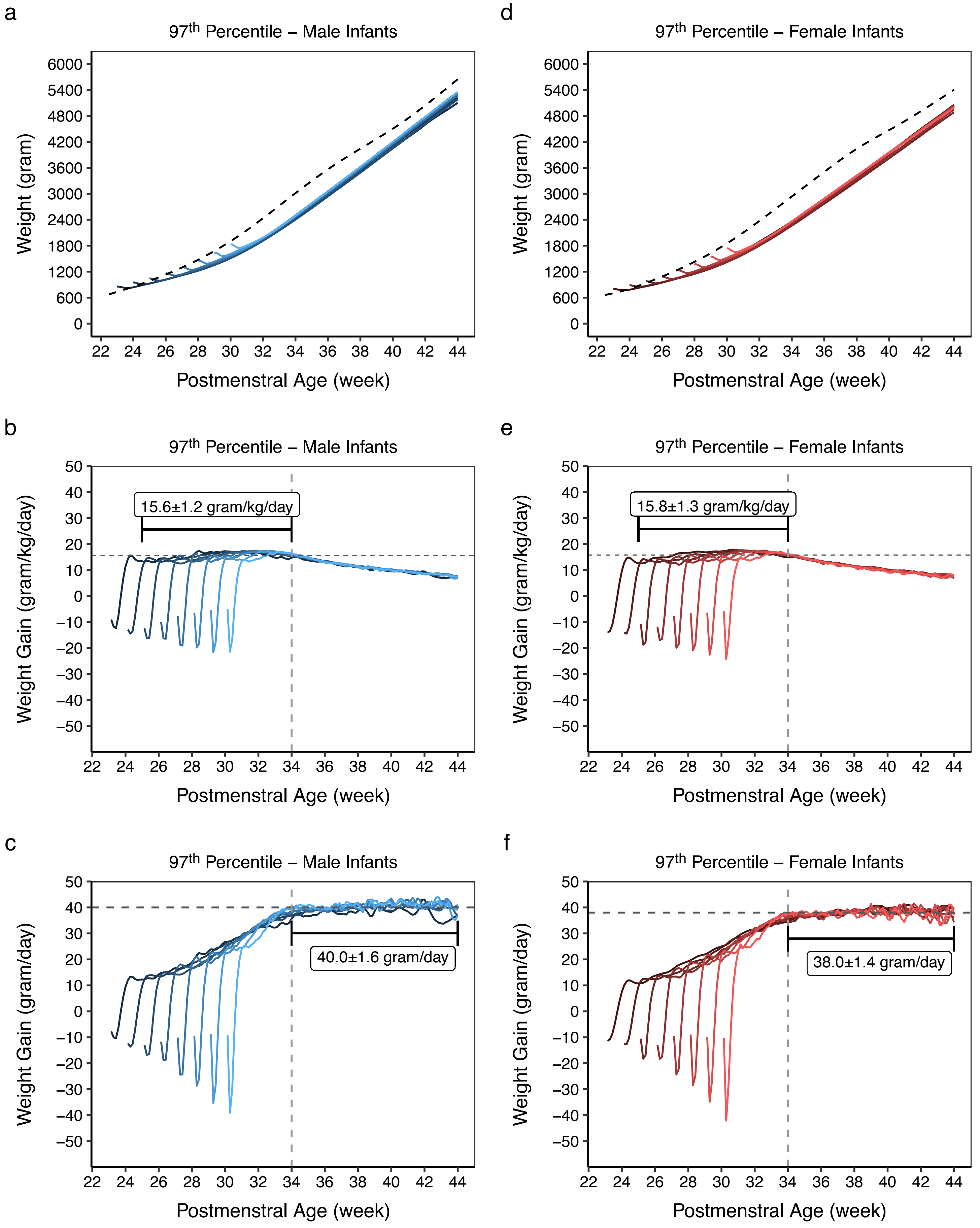

Figure S26. Sex differences in growth trajectory estimates. Postnatal growth models of (a) weight, (b) length, and (c) head circumference for male (blue) and female (red) infants of each gestational age group were plotted together to compare the trajectory estimates between the two sex groups. From top to down are 97th, 90th, 50th, 10th, and 3rd percentile lines.

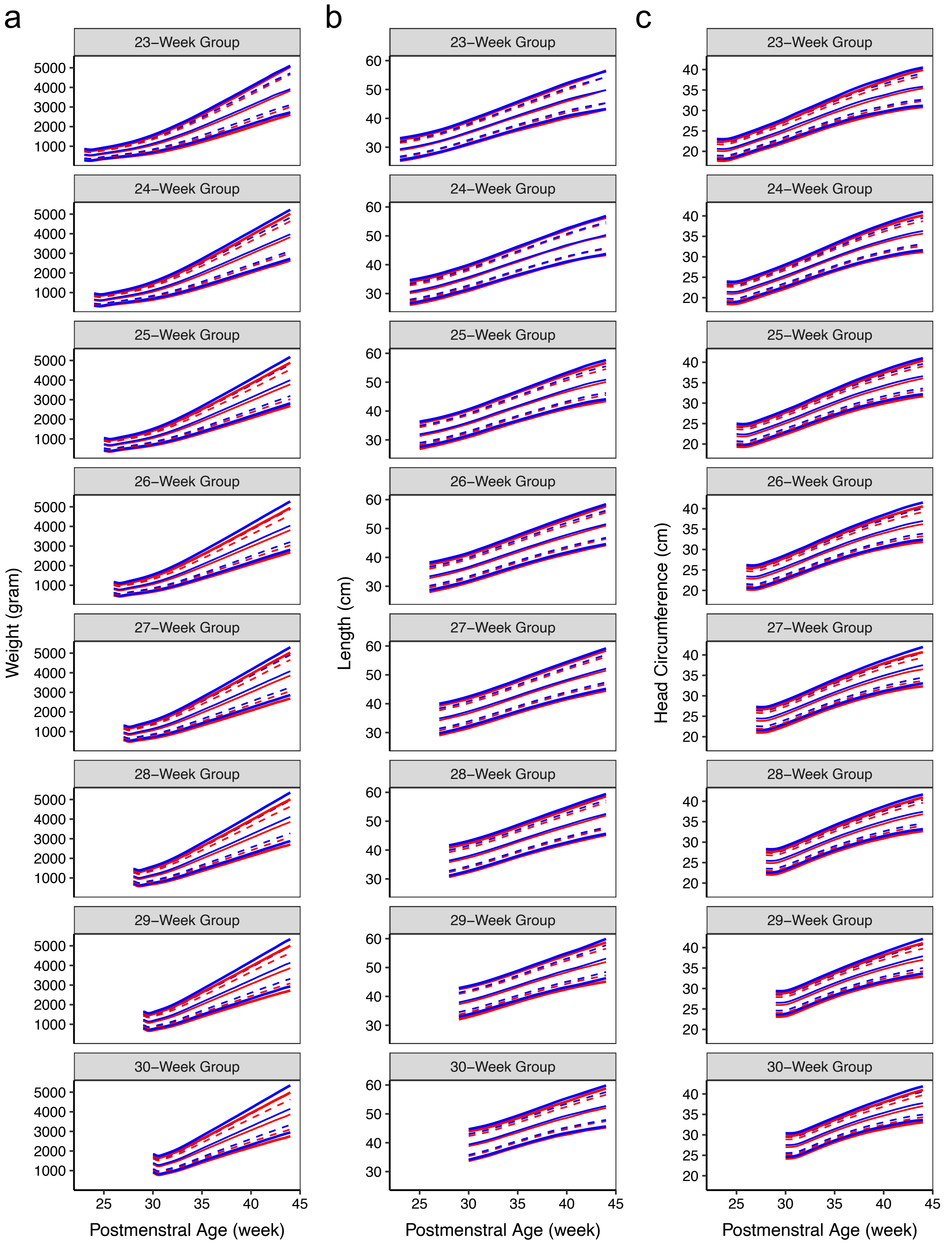

Supplement: Supplementary file 1 — Supplementary Information [file 41467_2023_41069_MOESM1_ESM.pdf]
