# Supplementary material for: A comparative study of postnatal anthropometric growth in very preterm infants and intrauterine growth
Source: Nat Commun. 2023 Sep 19;14:5626. doi: 10.1038/s41467-023-41069-0 (PMC10509139; doi:10.1038/s41467-023-41069-0)
Supplement: Supplementary file 3 — Description of Additional Supplementary Files [file 41467_2023_41069_MOESM3_ESM.pdf]

Description of Additional Supplementary Files

Supplementary Software File: The zipped file contains the comprehensive R codes (RScript.R) for postnatal growth trajectory modeling, variance and standard deviation calculation, and Percentile value calculation. The system requirement, instructions on running the R codes are provided in the README.txt file. A sample dataset (data.csv) is also included.
