# Supplementary material for: A comparative study of postnatal anthropometric growth in very preterm infants and intrauterine growth
Source: Nat Commun. 2023 Sep 19;14:5626. doi: 10.1038/s41467-023-41069-0 (PMC10509139; doi:10.1038/s41467-023-41069-0)
Supplement: Supplementary file 5 — Reporting Summary [file 41467_2023_41069_MOESM5_ESM.pdf]

nature portfolio

Corresponding author(s): Fu-Sheng Chou

Last updated by author(s): 7/5/2023

Reporting Summary

Nature Portfolio wishes to improve the reproducibility of the work that we publish. This form provides structure for consistency and transparency in reporting. For further information on Nature Portfolio policies, see our Editorial Policies and the Editorial Policy Checklist.

Statistics

For all statistical analyses, confirm that the following items are present in the figure legend, table legend, main text, or Methods section.

n/a	Confirmed	
☐	☒	The exact sample size (*n*) for each experimental group/condition, given as a discrete number and unit of measurement
☐	☒	A statement on whether measurements were taken from distinct samples or whether the same sample was measured repeatedly
☒	☐	The statistical test(s) used AND whether they are one- or two-sided<br>*Only common tests should be described solely by name; describe more complex techniques in the Methods section.*
☒	☐	A description of all covariates tested
☐	☒	A description of any assumptions or corrections, such as tests of normality and adjustment for multiple comparisons
☐	☒	A full description of the statistical parameters including central tendency (e.g. means) or other basic estimates (e.g. regression coefficient) AND variation (e.g. standard deviation) or associated estimates of uncertainty (e.g. confidence intervals)
☒	☐	For null hypothesis testing, the test statistic (e.g. *F*, *t*, *r*) with confidence intervals, effect sizes, degrees of freedom and *P* value noted<br>*Give P values as exact values whenever suitable.*
☒	☐	For Bayesian analysis, information on the choice of priors and Markov chain Monte Carlo settings
☐	☒	For hierarchical and complex designs, identification of the appropriate level for tests and full reporting of outcomes
☒	☐	Estimates of effect sizes (e.g. Cohen's *d*, Pearson's *r*), indicating how they were calculated

Our web collection on statistics for biologists contains articles on many of the points above.

Software and code

Policy information about availability of computer code

Data collection	Data were obtained from the Pediatrix® Clinical Data Warehouse (CDW), a large, multicenter, deidentified dataset that has been used in neonatal-perinatal research. The CDW is generated by a proprietary standardized documentation and billing software tool, BabySteps®, created by Pediatrix® and used by participating NICUs in 35 states and Puerto Rico.
Data analysis	R codes for postnatal growth modeling, total variance calculation, and percentile value calculation are available in the Supplementary Software file. R codes for plotting and for the WebApp are available on GitHub (https://github.com/fschou-pxt/GTC-Website-Apps.git).

For manuscripts utilizing custom algorithms or software that are central to the research but not yet described in published literature, software must be made available to editors and reviewers. We strongly encourage code deposition in a community repository (e.g. GitHub). See the Nature Portfolio guidelines for submitting code & software for further information.

Data

Policy information about availability of data

All manuscripts must include a data availability statement. This statement should provide the following information, where applicable:

- Accession codes, unique identifiers, or web links for publicly available datasets
- A description of any restrictions on data availability
- For clinical datasets or third party data, please ensure that the statement adheres to our policy

The postnatal growth curves of all gestational age groups are available publicly by accessing the WebApp (https://nicugrowth.app), without any limitation. Tabular

data of the postnatal growth curves developed in the current study cannot be shared openly due to concerns over the data being used for profit-generating purposes. Access can be obtained by sending a request via E-Mail to the corresponding author of the study (Dr. Fu-Sheng Chou, E-Mail: Fu-Sheng.X.Chou@kp.org), who will respond to the request within 7 days. A contract stating exclusive non-for-profit use by the requester, along with a signature, will be required for growth curve tabular data sharing. After receiving the document, Dr. Chou will share the data within 7 days. The authors do not have the permission to share the raw data supporting the current study due to the restriction of their contract with Pediatrix Medical Group Center for Research, Education, Quality, and Safety. Raw data can be requested by contacting either Dr. Fu-Sheng Chou (Fu-Sheng.X.Chou@kp.org) or Dr. Reese H. Clark (reese.clark@pediatrix.com), who will respond to the request within 7 days. Once the request is approved by the Pediatrix Medical Group Center for Research, Education, Quality, and Safety, Dr. Chou will share the data within 7 days. The authors received growth charts data pertaining to the 2013 Fenton growth charts from Dr. Tanis R. Fenton from the University of Calgary but were not given permission to publicly share these data. Access to these data can be addressed to Dr. Tanis R. Fenton (tfenton@ucalgary.ca) who will review the request for research or individual use only and share the data within a reasonable time frame (in our experience it was within 7 days). Further details may be obtained by contacting Dr. Fenton directly. The raw and summarized data generated in this study and presented in the main figures are provided in the Supplementary Information/Source Data file.

Research involving human participants, their data, or biological material

Policy information about studies with human participants or human data. See also policy information about sex, gender (identity/presentation), and sexual orientation and race, ethnicity and racism.

Reporting on sex and gender	The analyses were performed separately for the male and female sex groups.
Reporting on race, ethnicity, or other socially relevant groupings	The composition of the five-category racial/ethnic groups (White, Black, Hispanic, Asian, and Other) of the dataset is summarized in Table 1. The postnatal growth models were developed without excluding any racial/ethnic group(s).
Population characteristics	All infants with anthropometric records available between 2010 through 2020, regardless of being alive or dead, with a gestational age between 22 weeks 4 days and 30 weeks 3 days, a sex assigned based on physical features or genetic testing reports, and an admission age before day of life (DOL) 7 were included. Infants without sex assignment or without any measurement values available in the CDW were excluded. The presence or absence of antenatal exposure, congenital anomalies, multiple births, postnatal morbidities, or mortality were not used to determine infant inclusion or exclusion. Stillborn infants and infants who die in the delivery room are not admitted to the NICU and so are excluded from the CDW. All included infants had birth measurement data available, including birth weight, length, and head circumference. The electronic documentation tool does not have limits on birth measurement values.
Recruitment	All infants that met the gestational age and sex criteria with data available in the Clinical Data Warehouse were recruited.
Ethics oversight	The study was approved by the Research Advisory Committee of the Pediatrix® Center for Research, Education, Quality and Safety, and was exempted from the informed consent requirement by the Kaiser Permanente Southern California Institutional Review Board.

Note that full information on the approval of the study protocol must also be provided in the manuscript.

Field-specific reporting

Please select the one below that is the best fit for your research. If you are not sure, read the appropriate sections before making your selection.

☒ Life sciences ☐ Behavioural & social sciences ☐ Ecological, evolutionary & environmental sciences

For a reference copy of the document with all sections, see nature.com/documents/nr-reporting-summary-flat.pdf

Life sciences study design

All studies must disclose on these points even when the disclosure is negative.

Sample size	This is a large-scale growth charts study using real-world data compiled from clinical documentation. Sample size was determined based on the available infants in the database that met inclusion/exclusion criteria.
Data exclusions	No data was excluded.
Replication	Not applicable. This is a clinical observational study. There is only one dataset. All data from the dataset were used for model development.
Randomization	Not applicable. This is not a clinical trial study. There was intervention in study design. No randomization was needed.
Blinding	Not applicable. This is a clinical observational study. There was no intervention in study design. Therefore, blinding was not necessary.

Reporting for specific materials, systems and methods

We require information from authors about some types of materials, experimental systems and methods used in many studies. Here, indicate whether each material, system or method listed is relevant to your study. If you are not sure if a list item applies to your research, read the appropriate section before selecting a response.

Materials & experimental systems

n/a	Involved in the study
☒	☐ Antibodies
☒	☐ Eukaryotic cell lines
☒	☐ Palaeontology and archaeology
☒	☐ Animals and other organisms
☒	☐ Clinical data
☒	☐ Dual use research of concern
☒	☐ Plants

Methods

n/a	Involved in the study
☒	☐ ChIP-seq
☒	☐ Flow cytometry
☒	☐ MRI-based neuroimaging
